# Physiologically mediated responses in gilthead sea bream (*Sparus aurata*) fed sustainable diets: seasonal growth under warming conditions

**DOI:** 10.3389/fphys.2026.1860904

**Published:** 2026-06-30

**Authors:** Paul George Holhorea, Federico Moroni, Itziar Estensoro, Alvaro Belenguer, Ricardo Domingo-Bretón, Fernando Naya-Català, Josep Calduch-Giner, Jaume Pérez-Sánchez

**Affiliations:** 1 Nutrigenomics and Integrative Fish Biology Group, Institute of Aquaculture Torre de la Sal (IATS, CSIC), Castellón, Spain; 2 Pathology Group, Institute of Aquaculture Torre de la Sal (IATS, CSIC), Castellón, Spain

**Keywords:** alternative protein sources, behavioral coping, climate-resilient aquaculture, growth performance, physiological plasticity, thermal tolerance

## Abstract

**Background:**

The Mediterranean Sea is warming approximately 20% faster than the global average, underscoring the urgent need for adaptive aquaculture strategies, including the evaluation of novel feed formulations under environmentally relevant conditions. To address this objective, the present study assessed the performance of gilthead sea bream during the unusually warm summers recently recorded in the Western Mediterranean, where temperatures have increased by approximately 0.1 °C per year over the past decade. The rising temperatures between 2020 and 2025 were associated with progressively shorter production cycles, particularly in the 2022 cohort, where growth rates increased by approximately 10-15%.

**Methods:**

In this cohort, we conducted a ten−month trial (May 2022–February 2023) to evaluate juvenile performance using a plant−based diet representative of current commercial formulations, along with two experimental diets in which fishmeal and fish oil were partially replaced by either processed animal proteins and salmon oil by−products (PAP diet) or a combination of insect proteins, microbial biomass, and algal oil (ALT diet).

**Results:**

Growth performance remained largely comparable across dietary treatments, and histological analyses of liver and anterior/posterior intestine at three sampling points (July, November, February) revealed no tissue alterations, indicating preserved organ integrity throughout the trial. Despite similar growth outcomes, fish relied on distinct physiological strategies to sustain performance. The ALT diet was associated with reduced basal stress−axis activity, more proactive behavioral responses following confinement, and enhanced intestinal transcriptional activity, suggesting improved physiological responses to aquaculture stressors. Conversely, the PAP diet was associated with a down-regulation of liver and white muscle transcriptomes, which may indicate a potential trade−off that could affect cold−adaptation capacity.

**Conclusion:**

Overall, these findings suggest that alternative and low−trophic feed ingredients following circular economy principles can sustain growth and tissue health even under atypically warm conditions, while also modulating stress physiology in ways that may enhance robustness in a rapidly warming Mediterranean. By identifying diet−specific physiological signatures, this study provides actionable insights for the design of aquafeeds better adapted to warming conditions, supporting the transition toward more sustainable and adaptive aquaculture systems, particularly in the Mediterranean region, where accelerating warming demands rapid innovation to maintain productivity, animal welfare, and long-term sector sustainability.

## 1 Introduction

Aquaculture has become the fastest-growing food production sector, with an average annual growth of 6.5%, excluding the algae production (FAO, 2022). However, its long-term sustainability is increasingly challenged by climate change, a global driver with significant impacts on ecosystems and human societies (Cramer et al., 2018). Certainly, over the past 120 years, the sea surface temperature (SST) has increased continuously (0.062 ± 0.013 °C per decade), but this warming rate has been accelerated more than four times between 2010 and 2019 (García-Soto et al., 2021). The Mediterranean Sea is particularly susceptible to this general warming due to its semi-enclosed configuration, which allowed an increased SST temperature three times greater than open oceans over the last 40 years (Pisano et al., 2020; IPCC Core Writing Team, 2023). This situation represents a serious risk for Mediterranean aquaculture with increasing frequency and intensity of marine heatwaves (Dayan et al., 2023; Hamdeno & Alvera-Azcaráte, 2023; Atalah et al., 2024), indicating the predictive models that prolonged thermal stress periods would significantly affect the efficiency and profitability of the sector (Haberle et al., 2024). Therefore, the adaptation to climate change is crucial for the sustainable development of the Mediterranean aquaculture. Possible solutions include the adoption of more appropriate management and nutritional solutions, a switch to more heat-tolerant strains and fish species, and the reallocation of production to the Western Mediterranean to ensure better water quality and reduced thermal variability for commonly farmed species such as gilthead sea bream (Sparus aurata) and European sea bass (Dicentrarchus labrax), but also for emerging species including meagre (Argyrosomus regius) and greater amberjack (Seriola dumerili) (Islam et al., 2021; Stavrakidis-Zachou et al., 2021; Abdel-Tawwab et al., 2025; Carvalheiro et al., 2025; Navarro-Guillén et al., 2025).

At the cellular and organism level, fish respond to thermal stress through a suite of physiological and behavioral mechanisms that activate protective and antioxidant pathways, alter activity patterns or habitat use, and, over longer timescales, enable adaptation through generational change (Currie et al., 2014; Farrell et al., 2009; Alfonso et al., 2021; Vargas-Chacoff et al., 2020; Petitjean et al., 2019; Volkoff and Rønnestad, 2020; Sandoval-Castillo et al., 2020; Roy et al., 2023). Therefore, understanding these multilevel responses is essential for anticipating how wild and farmed fish in particular will cope with the increasing occurrence of thermal anomalies. In the case of gilthead sea bream, early studies have evidenced that short-term exposure to temperatures above 28 °C (< 2 weeks) induces tissue damage, impaired immunity and increased oxidative stress, temporarily compromising growth and overall performance (Madeira et al., 2016; Kir, 2020; Zarantoniello et al., 2021). Such elevated temperatures also disrupt gut homeostasis, reflected by an increased abundance of *Brevinema*, a microbial biomarker of heat stress. However, dietary interventions, such as emulsifier-supplemented and low-calorie diets can largely mitigate this microbial imbalance (Domingo-Breton et al., 2025a). In the same way, rapid thermal ramps disrupt musculoskeletal development and lipid metabolism, further evidencing potential vulnerabilities under abrupt temperature increases (Balbuena-Pecino et al., 2019; Madeira et al., 2016). Taken together, these findings suggest that gilthead sea bream has a limited capacity to acclimatize to temperature variations between 26 °C and 30 °C, although some evidence indicates that thermal tolerance during the grow-out phase may be higher than previously assumed (Kourkouta et al., 2021; Almeida et al., 2025). In parallel, extensive evidence shows that not only plant-based ingredients, but also other alternative raw materials, can be effectively incorporated into low- or fishmeal (FM)-free diets when formulations are designed to meet species-specific nutritional requirements (Alfonso et al., 2024; Fernandes et al., 2024; Serra et al., 2024; Mendes et al., 2025). Nevertheless, despite their demonstrated efficacy under standard conditions, the feasibility of these novel feed formulations in the context of global warming remains largely uncertain. Addressing this knowledge gap is therefore essential not only to reduce reliance on traditional fish feed ingredients, but also to enhance the adaptive capacity and welfare of gilthead sea bream and other Mediterranean aquaculture species facing increasingly frequent thermal challenges.

Building on the potential of new fish feeds for Mediterranean fish species, the present study aimed to evaluate the impact of novel fish feeds for gilthead sea bream under conditions relevant to climate change. In particular, we examined whether growth performance under warming conditions is associated with different physiological adaptation strategies depending on diet formulation. Diets incorporating salmon oil-by product and processed animal proteins (PAPs), as well as algae oil, microbial biomass and insect proteins were assessed in a long-term trial for their effects on growth performance and gene expression in metabolically (liver, skeletal muscle) and immunologically (intestine) relevant tissues. Signs of histopathological damage were also assessed in liver and anterior/posterior intestine sections. All this forms part of a more comprehensive study within the framework of the Next Generation EU project (GVA-ThinkInAzul), which also considered the effects of new diet formulations and climate-change-relevant conditions on food safety (González-Hernández et al., 2025) and quality (Jiménez-Redondo et al., 2025). Additional studies are also currently underway to comprehensively evaluate the changing water and mucosal microbiome of gilthead sea bream as a unique ecosystemic unit (Domingo-Breton et al., 2024; Domingo-Breton et al., 2025b).

## 2 Materials and methods

### 2.1 Ethics statement

All procedures were approved by the Ethics and Animal Welfare Committee of the Institute of Aquaculture Torre de la Sal (IATS, CSIC), CSIC Ethics Comittee (permissions 1295/2022) and Generalitat Valenciana (permission 2022-VSC-PEA-0230). They were carried out in the IATS’s registered aquaculture infrastructure facility (code ES120330001055) in accordance with the principles published in the European Animal Directive (2010/63/EU) and Spanish laws (Royal Decree RD53/2013) for the protection of animals used in scientific experiments.

### 2.2 Diets

Three experimental extruded diets, with protein and lipid contents adjusted according to fish growth, were manufactured by Sparos Lda. (Portugal). For each pellet-size class (i.e., 2 and 3–4.5 mm), diets were formulated to provide comparable protein (2 mm: 49.9 ± 0.7% DM; 3–4.5 mm: 48.3 ± 0.1% DM) and lipid levels (2 mm: 18.0 ± 0.2% DM; 3–4.5 mm: 18.7 ± 0.4% DM) while satisfying the species-specific requirements for essential amino acids (Kaushik, 1998; Peres and Oliva-Teles, 2009) and long-chain polyunsaturated fatty acids, including EPA and DHA levels above the 0.9% requirement reported for gilthead sea bream juveniles (Kalogeropoulos, 1992). The control diet (CTRL) mimicked current commercial plant-based formulations, with fish oil (FO; 5.4–6.6%) and fishmeal (FM; 5–17%) included as the main dietary lipid and protein sources, respectively. The two experimental diets were either partially (2 mm pellets) or completely (3–4.5 mm pellets) devoid of FM. In the PAP diet, poultry meal was used as the main additional FM replacer, whereas in the ALT diet, FM was further replaced by insect protein and microbial biomass. Otherwise, salmon-oil by-product and DHA algae oil were used as alternative oils in PAP and ALT diets, respectively. Experimental diets formulations and analyzed composition are shown in **Table 1**. Dry matter was measured by oven−drying at 105 °C for 24 h, and crude protein (N × 6.25) by the Kjeldahl method. Crude fat was quantified after dichloromethane extraction by the Soxhlet method, and crude fiber was estimated gravimetrically after acid and alkali digestion followed by combustion at 600 °C for 3 h. For fatty acid profiling, diet samples were subjected to *in situ* methylation following a two−step transesterification protocol consisting of a basic phase with sodium methoxide and a subsequent acid−catalyzed methylation with boron trifluoride in methanol. The resulting fatty acid methyl esters (FAMEs) were extracted into hexane and analyzed by GC−FID using a CP−Sil 88 capillary column. Fatty acids were identified by comparison with certified FAME standards.

**Table 1:** Ingredients and analyzed chemical composition of experimental diets with a pellet size of 2-, 3- and 4.5-mm. Bold values refer to the 2-mm pellet, red values to the 3- and 4.5-mm pellets; single values apply to all pellet sizes.

Ingredient (%)			
	CTRL	PAP	ALT
Fishmeal Super Prime	**17** – 5	0	0
Fishmeal 60	**5** – 10	**5** – 0	**5** – 0
Fish protein hydrolysate	**3** – 0	**3** – 0	**3** – 0
Poultry meal	12	**25** – 17	12
Porcine blood meal	5	8	5
Feathermeal hydrolysate	**5** – 7.25	**5** – 9.75	**5** – 7.25
Insect meal (PROTE – IN HP55)	0	0	**12** – 8.5
Aminopro NT70	0	0	**6** – 5
Corn gluten meal	**7** – 8	**7** – 8	**7** – 8
Soybean meal 44	**9** – 12	**9** – 12	**9** – 12
Sunflower meal 40	**4.5** – 7.5	**4.5** – 7.5	**4.5** – 7.5
Wheat meal	**11.28** – 9.58	**11.73** – 11.58	**8.88** – 8.88
Whole peas	5	5	5
Pea starch (raw)	2	2	2
Vitamin and mineral premix^1^	1	1	1
Vitamin E50	**0.**1	0.1	0.1
Antioxidant	0.2	0.2	0.2
Sodium propionate	0.1	0.1	0.1
MAP (Monoamonium phosphate)	**0.5** – 0.8	**0.75** – 1.9	**1.55** – 2.1
L – Lysine HCl 99%	**0** – 0.4	**0** – 0.6	**0.3** – 0.7
DL – Methionine	0	**0** – 0.1	**0** – 0.1
Yttrium oxide	0.02	0.02	0.02
Fish oil	**5.4** – 6.65	**5.4** – 6.65	**5.4** – 6.65
Salmon oil by-product	0	**7.2** – 3	0
Algae oil (Veramaris)	0	0	**0.9** – 0.4
Rapeseed oil	**6.9** – 7.4	**0** – 5.5	**6.05** – 7.5
Analyzed composition			
Dry matter (DM, %)	**95.4** – 94.0	**93.7** – 95.4	**94.5** – 93.4
Crude protein (% DM)	**50.3** – 48.2	**51.0** – 48.4	**48.5** – 48.1
Crude fat (% DM)	**17.7** – 17.9	**18.3** – 19.0	**18.1** – 19.2
Crude fiber (% DM)	**1.4** – 1.9	**1.4** – 1.9	**1.7** – 2.3
EPA + DHA (% DM)	**1.2** – 1.5	**1.4** – 1.9	**1.3** – 1.7

^1^ Vitamin and mineral premix: Vitamins (IU or mg/kg diet): DL - alpha tocopherol acetate, 565 mg; sodium menadione bisulphate, 23.5 mg; retinyl acetate, 21,750 IU; DL - cholecalciferol, 4,640 IU; thiamine, 28.3 mg; riboflavin, 28.6 mg; pyridoxine, 22 mg; cyanocobalamin, 0.1 mg; nicotinic acid, 205 mg; folic acid, 14 mg; ascorbic acid, 935 mg; inositol, 465 mg; biotin, 2.9 mg; calcium pantothenate, 95.5 mg; choline chloride, 1070 mg; betaine, 465 mg. Mineral (g or mg/kg diet): Na, 3 g; Mg, 1.4 g; K, 5.9 g; Cu, 15.6 mg; Fe, 240 mg; I, 0.95 mg; Mn, 19.9 mg; Se, 0.5 mg; Zn, 48 mg.

### 2.3 Multiple-diet feeding trial, sample collection and histological evaluation

Gilthead sea bream of Mediterranean origin (Avramar, Burriana, Spain) were reared from early life stages (10–15 g) in a flow-through system using duplicate-triplicate 3,000 L tanks (440–130 fish per tank, with fish progressively redistributed to maintain appropriate stocking densities according to animal growth) under natural photoperiod and temperature conditions at the IATS facility (latitude 40°5′ N, longitude 0°10′ E) from May 2022 to February 2023, with the three experimental diets (Section 2.2). The trial was then extended until May 2023, following the same feeding plan, for a complementary behavioural assessment (Section 2.6). Fish were fed twice a day (9:00 and 15:00 h) with 2/3/4.5 mm pellet size using automated feeders adjusted near to visual satiety. Unionized ammonia remained below 0.02 mg/L, and water O_2_ concentration was maintained higher than 75% saturation over the whole experimental period. Fish behaviour was daily monitored using high-quality top tank video cameras, and stocking density was always kept below 16 kg/m^3^ to ensure optimal welfare and maximize growth performance (Holhorea et al., 2023a).

At regular monthly intervals, overnight-fasted fish were anesthetized with 0.1 g/L MS-222 (Sigma, Saint Louis, MO, USA), and fish body weight and furcal length were measured individually using a FR-200 FishReader W (Trovan, Madrid, Spain). Blood and tissue samples were collected at three sampling times: T_1_, July-2022; T_2_, November-2022; T_3_, February-2023. At each sampling point, blood from anaesthetized fish (12–16 per experimental condition and sampling time) was taken from the caudal vessels using heparinized syringes. It was centrifuged at 3,000 x *g* for 20 min at 4°C, and plasma aliquots were stored at -20°C until biochemical analyses of cortisol and circulating metabolites. Dorsal muscle fat content was determined *in situ* with Distell Fish Fat-meter, FM 692 (Distell Ltd., United Kingdom) by four consecutive readings across the body to account for fat distribution variations. These fish were then killed by cervical section, and the liver and viscera weights were registered for calculating the hepatosomatic (HSI) and viscerosomatic (VSI) indices. Tissue portions (150–200 mg) of dorsal white skeletal muscle (WSM), liver, and anterior intestine (AI, 1–2 cm from the pyloric region) were excised and collected in RNA later for its storage at -80°C until gene expression analyses.

Additionally, portions of liver and AI were collected in buffered formalin (pH=7.2) at all time points and of posterior intestine (PI, 2–3 cm from the anal ampoule) at T_2_ and T_3_. Tissues were stored at 4 °C until further processing for paraffin histology and finally, 4 µm sections stained with Giemsa or with periodic acid-Schiff (PAS) were evaluated with a Leitz Dialux 22 light microscope and representative microphotographs taken with an Olympus DP70 camera. Histological alterations were registered according to a semiquantitative scoring scale (from 0 = absence to 3 = very abundant/severe). In intestinal sections, inflammatory markers (i.e., abundance of intraepithelial lymphocytes, abundance of eosinophilic granular cells, degree of submucosal hyperplasia), goblet cell abundance and degree of lipid vacuolization in enterocytes were quantified. In livers, the degree of lipid and glycogen storage in hepatocytes was scored.

### 2.4 Multi-year assessment of growth performance

To provide broader context for the multiple-diet feeding trial (Section 2.3), a complementary single-site longitudinal assessment of growth performance was conducted using records from five consecutive grow-out production cycles (2020–2025), using approximately 450–150 fish each, carried out from March/May to January/February at the IATS-CSIC. This analysis included data from fish fed standard commercial diets (Inicio Plus M; Intro Plus MT; EFICO 3053; BioMar, Palencia, Spain) in addition to those of CTRL fish from the multiple-diet feeding trial conducted during the 2022 production cycle. All fish stocks shared the same genetic background and were reared under equivalent husbandry conditions in 3,000 L tanks, allowing performance comparisons across years. Growth trajectories were assessed by measures of specific growth rates (SGR) and thermal-unit growth coefficient (TGC) to produce an approximately constant index within a given growth stanza and temperature. To characterize the environmental context, water temperature was continuously recorded at 5−min intervals, and a daily mean temperature time series for 2020–2025 was constructed (**Supplementary Figure 1**), capturing the characteristic seasonal cycle with winter minima (January-February) and summer peaks (August). For a long-term thermal context, daily mean temperature data (IATS facilities) from 2013-2023 (**Supplementary Table 1**) were used to estimate annual and seasonal trends. The average annual temperature over this decade was used as a reference threshold to quantify the number of days exceeding this value during each cycle from 2020-2025, allowing assessment of interannual warming intensity and its potential influence on growth.

### 2.5 Biochemical and molecular analyses

Plasma glucose was determined using the Invitrogen™ Glucose Colorimetric Detection Kit (Invitrogen, EIAGLUC, Carlsbad, CA, USA). Plasma cortisol levels were determined with an enzyme Immunoassay Kit (Arbor Assays, K003-H1W, Ann Arbor, MI, USA) following the manufacturer’s indications. Total plasma cholesterol was determined using a cholesterol esterase/cholesterol dehydrogenase reagent (ThermoFisher Scientific, Middletown, VA, USA). Triglycerides (TG) were analysed using a commercial kit (981786, ThermoFisher Scientific, Vantaa, Finland).

Tissue RNA was extracted using the MagMAX-96 total RNA isolation kit (Life Technologies, Carlsbad, CA, USA) after tissue homogenization in TRI reagent following manufacturers’ instructions. RNA quantity and purity was determined by Nanodrop (Thermo Scientific, Waltham, MA, USA) with absorbance ratios at 260 nm/280 nm of 1.9-2.1. Reverse transcription (RT) of 500 ng of total RNA was performed with random decamers using the High-Capacity cDNA Archive Kit (Applied Biosystems, Foster City, CA, USA). RT reactions were incubated for 10 min at 25 °C and 2 h at 37 °C. Negative control reactions were run without reverse transcriptase. Real-time quantitative PCR was carried out with a CFX96 Connect™ Real-Time PCR Detection System (Bio-Rad, USA), using 96-well PCR array layouts designed for the simultaneous profiling of 44–38 selected genes (108 genes in total) of liver, WSM and AI using customized tissue-specific PCR-arrays (**Tables 2**, **3**). The genes comprised in the liver array included markers of the growth hormone (Gh) and the insulin-like growth factor (Igf) system (9), lipid metabolism (14), oxidative metabolism and energy sensing (12), and antioxidant defence (9). The analysed transcripts of WSM included markers of the Gh/Igf system (6), muscle cell growth (7), oxidative metabolism and energy sensing (10), antioxidant defence (7) and protein breakdown (8). Specific primer pair sequences for liver and WSM are listed in **Supplementary Table 2**. The genes comprised in the AI array included markers of epithelial integrity (11), mucus production (2), nutrient transport (4), cytokines and chemokine-related proteins (13), T cell and monocyte/macrophage markers (4), pattern recognition receptors (8) and immunoglobulins (2). Specific primer pair sequences for AI are listed in **Supplementary Table 3**.

**Table 2:** PCR-array layout for liver (*) and white skeletal muscle (**^†^**) gene expression profiling.

Function	Gene	Symbol	GenBank
GH/IGF system	Growth hormone receptor-type 1	ghr1*^†^	AF438176
	Growth hormone receptor-type 2	ghr2*^†^	AY573601
	Insulin-like growth factor 1	igf1*^†^	AY996779
	Insulin-like growth factor 2	igf2*^†^	AY996778
	Insulin-like growth factor binding protein 1a	igfbp1a*	KM522771
	Insulin-like growth factor binding protein 1b	igfbp1b*	MH577189
	Insulin-like growth factor binding protein 2a	igfbp2a*	MH577190
	Insulin-like growth factor binding protein 2b	igfbp2b*	AF377998
	Insulin-like growth factor binding protein 3a	*igfbp3a* ^†^	MH577191
	Insulin-like growth factor binding protein 4	igfbp4*	KM658998
	Insulin-like growth factor binding protein 5b	*igfbp5b* ^†^	MH577194
Lipid metabolism	Elongation of very long chain fatty acids 1	elovl1*	JX975700
	Elongation of very long chain fatty acids 4	elovl4*	JX975701
	Elongation of very long chain fatty acids 5	elovl5*	AY660879
	Elongation of very long chain fatty acids 6	elovl6*	JX975702
	Fatty acid desaturase 2	fads2*	AY055749
	Stearoyl-CoA desaturase 1a	scd1a*	JQ277703
	Stearoyl-CoA desaturase 1b	scd1b*	JQ277704
	Hepatic lipase	hl*	EU254479
	Lipoprotein lipase	lpl*	AY495672
	Adipose triglyceride lipase	atgl*	JX975711
	85kDa calcium-independent phospholipase A2	pla2g6*	JX975708
	Cholesterol 7-alpha-monooxygenase	cyp7a1*	KX122017
	Peroxisome proliferator-activated receptor α	pparα*	AY590299
	Peroxisome proliferator-activated receptor γ	pparγ*	AY590304
Muscle cell growth	Myoblast determination protein 1	*myod1* ^†^	AF478568
	Myogenic determination protein 2	*myod2* ^†^	AF478569
	Myogenic factor 5	*myf5* ^†^	JN034420
	Myogenic factor 6	*myf6/mrf4* ^†^	JN034421
	Myostatin/Growth differentiation factor 8	*mstn* ^†^	AF258448
	Follistatin	*fst* ^†^	AY544167
	Cadherin 15	*cdh15* ^†^	KM522781
Oxidative metabolism & energy sensing	Hypoxia inducible factor 1*α*	hif1α*^†^	JQ308830
	Proliferator-activated receptor γ coactivator 1*α*	pgc1α*	JX975264
	Carnitine palmitoyltransferase 1a	cpt1a*^†^	JQ308822
	Fatty acid binding protein, heart	hfabp*	JQ308834
	Citrate synthase	cs*^†^	JX975229
	NADH-ubiquinone oxidoreductase chain 2	nd2*^†^	KC217558
	NADH-ubiquinone oxidoreductase chain 5	nd5*^†^	KC217559
	Cytochrome c oxidase subunit 1	cox1*^†^	KC217652
	Cytochrome c oxidase subunit 2	cox2*^†^	KC217653
	Uncoupling protein 1	ucp1*	FJ710211
	Uncoupling protein 3	*ucp3* ^†^	EU555336
	Sirtuin1	sirt1*^†^	KF018666
	Sirtuin2	sirt2*^†^	KF018667
Antioxidant defence	Glutathione peroxidase 1	gpx1*	DQ524992
	Glutathione peroxidase 4	gpx4*^†^	AM977818
	Glutathione reductase	*gr* ^†^	AJ937873
	Peroxiredoxin 3	prdx3*	GQ252681
	Peroxiredoxin 5	prdx5*	GQ252683
	Superoxide dismutase [Cu-Zn]	cu-zn-sod / sod1*	JQ308832
	Superoxide dismutase [Mn]	mn-sod / sod2*^†^	JQ308833
	Glucose-regulated protein 170 kDa	grp170*^†^	JQ308821
	Glucose-regulated protein 94 kDa	grp94*^†^	JQ308820
	Glucose-regulated protein 75 kDa	grp75*^†^	DQ524993
	Catalase	*cat* ^†^	JQ308823
Protein breakdown	Calpain 1	*capn1* ^†^	KF444899
	Calpain 2	*capn2* ^†^	KF444900
	Calpain 3	*capn3* ^†^	KM522785
	Calpastatin	*cast* ^†^	KM522786
	Cathepsin B	*ctsb* ^†^	KJ524457
	Cathepsin D	*ctsd* ^†^	AF036319
	Cathepsin L	*ctsl* ^†^	KM522787
	Cathepsin S	*ctss* ^†^	KM522788

**Table 3:** PCR-array layout for anterior intestine gene expression profiling.

Function	Gene	Symbol	GenBank
Epithelial integrity	Proliferating cell nuclear antigen	*pcna*	KF857335
	Transcription factor HES-1-B	*hes1b*	KF857344
	Krueppel-like factor 4	*klf4*	KF857346
	Claudin-12	*cldn12*	KF861992
	Claudin-15	*cldn15*	KF861993
	Cadherin-1	*cdh1*	KF861995
	Cadherin-17	*cdh17*	KF861996
	Tight junction protein ZO-1	*tjp1*	KF861994
	Desmoplakin	*dsp*	KF861999
	Gap junction Cx32.2 protein	*cx32.2*	KF862000
	Coxsackievirus and adenovirus receptor homolog	*cxadr*	KF861998
Mucus production	Mucin 2	*muc2*	JQ277710
	Mucin 13	*muc13*	JQ277713
Nutrient transport	Intestinal-type alkaline phosphatase	*alpi*	KF857309
	Liver type fatty acid-binding protein	*fabp1*	KF857311
	Intestinal fatty acid-binding protein	*fabp2*	KF857310
	Ileal fatty acid-binding protein	*fabp6*	KF857312
Cytokines and chemokine-related proteins	Tumor necrosis factor alpha	*tnfα*	AJ413189
	Interleukin-1 beta	*il1β*	AJ419178
	Interleukin-6	*il6*	EU244588
	Interleukin-7	*il7*	JX976618
	Interleukin-8	*il8*	JX976619
	Interleukin-10	*il10*	JX976621
	Interleukin-12 subunit beta	*il12 β*	JX976624
	Interleukin-15	*il15*	JX976625
	Interleukin-34	*il34*	JX976629
	C-C chemokine receptor type 3	*ccr3*	KF857317
	C-C chemokine receptor type 9	*ccr9*	KF857318
	C-C chemokine receptor type 11	*ccr11*	KF857319
	C-C chemokine CK8 / C-C motif chemokine 20	*ck8/ccl20*	GU181393
T cell and monocyte / macrophage markers	Cluster of differentiation 4	*cd4-1*	AM489485
	Cluster of differentiation 8 beta	*cd8b*	KX231275
	Macrophage colony-stimulating factor 1 receptor 1	*csf1r1*	AM050293
	Macrophage mannose receptor 1	*mrc1*	KF857326
Pattern recognition receptors (PRRs)	Galectin-1	*lgals1*	KF862003
	Galectin-8	*lgals8*	KF862004
	Toll-like receptor 2	*tlr2*	KF857323
	Toll-like receptor 5	*tlr5*	KF857324
	Toll-like receptor 9	*tlr9*	AY751797
	CD209 antigen-like protein D	*cd209d*	KF857327
	CD302 antigen	*cd302*	KF857328
	Fucolectin	*fcl*	KF857331
Immunoglobulins	Immunoglobulin M	*igm*	JQ811851
	Immunoglobulin T membrane-bound form	*igt-m*	KX599201

Controls of general PCR performance were included on each PCR-array, and all the pipetting operations were performed by means of an EpMotion 5070 Liquid Handling Robot (Eppendorf, Hamburg, Germany) to improve data reproducibility. Briefly, reverse transcription reactions were diluted to convenient concentrations and the equivalent of 660 pg of total input RNA was used in a 25 μL volume for each PCR reaction. PCR-wells contained a 2× SYBR Green Master Mix (Bio-Rad, Hercules, CA, USA), and specific primers at a final concentration of 0.9 μM were used to obtain amplicons of 50–150 bp in length. The PCR amplification program consisted of an initial denaturation step at 95 °C for 3 min, followed by 40 cycles of denaturation for 15 s at 95 °C and annealing/extension for 60 s at 60 °C. The expression stability of four candidate housekeeping genes (β-actin, elongation factor 1α, α−tubulin and 18S rRNA) was evaluated using the geNorm algorithm (version 3.5). Based on their stability values across tissues, sampling times and dietary treatments, β−actin (M score = 0.18-0.23) and elongation factor−1α (M score = 0.21-0.27) were selected as the reference pair, and qPCR data were normalized to the geometric mean of these two genes. The efficiency of PCR reactions for all target and reference genes varied between 91% and 99% (**Supplementary Tables 2**, **3**), and reaction specificity was verified by melting curve analysis (ramping rates of 0.5 °C/10 s over a temperature range of 55-95 °C) and by assessing the linearity of serial dilutions of RT reactions. The dynamic range of standard curves spanned five orders of magnitude, and the amount of product in each sample was calculated by interpolation of the corresponding C_t_ value. Gene expression levels were calculated using the delta-delta C_t_ method (Livak and Schmittgen, 2001). For multigene expression analysis, all values in the liver and WSM were referenced to the expression levels of *ghr1* in CTRL_T_1_ fish with an arbitrary assigned value of 1. In the case of the AI, all values were referenced to the expression levels of *ccr9* in CTRL_T_1_ fish with an arbitrary assigned value of 1.

### 2.6 Behavioral monitoring: stress test

**Figure 1:**
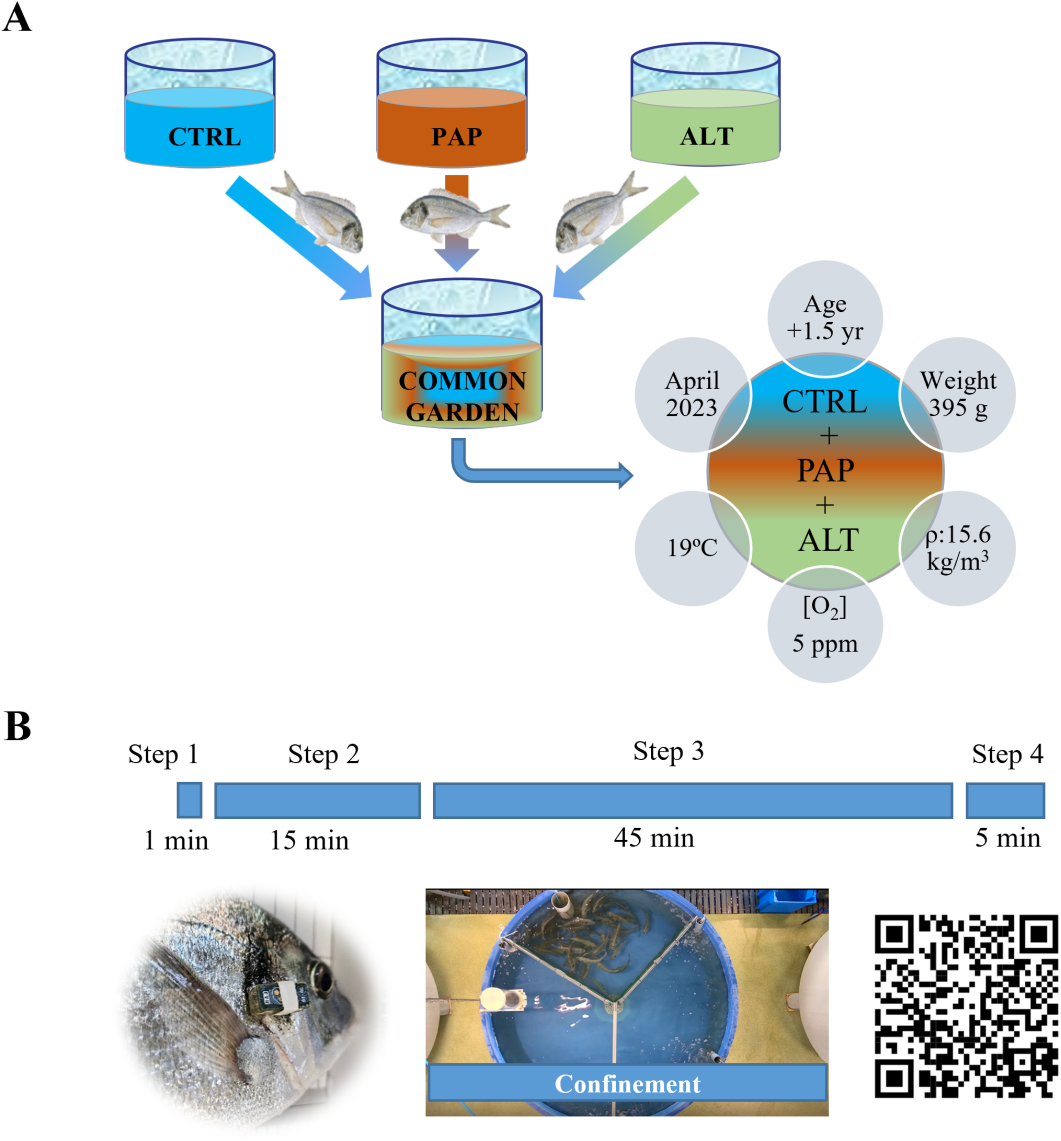
Experimental setup of the confinement stress test. **(A)** Explanatory illustration of the fish involved in the stress test. **(B)** Confinement method explained in four steps; Step 1 (Structure insertion), Step 2 (Folded structure), Step 3 (Unfolded structure and fish compression), Step 4 (Folded structure and removal). Video of the process is available scanning the QR code.

In May 2023, three months following the extension of the initial feeding trial (May 2022-February 2023), a total of 37 fish per dietary condition were randomly selected, anaesthetized with a 0.1 g/L MS-222 solution (Sigma, Saint Louis, MO, USA), and housed together (common garden system) in a flow-through 3,000 L tank (**Figure 1A**). At the same time, the AEFishBIT device was attached externally to the operculum of six fish per dietary group for the simultaneous monitoring of both physical activity and respiratory frequency as reviewed by Calduch-Giner et al. (2022). One day thereafter, fish with an averaged body weight of 395 g were subjected to a standardized and reproducible confinement stress test carried out at 19 °C with a water O_2_ concentration above 70% saturation (Holhorea et al., 2024). Briefly, the stress test consisted in placing a folded self-made PVC confinement structure into the tank for 15 min, allowing fish to become acclimated to the new object. Subsequently, the structure was un-folded, and fish were confined to one portion of the tank for a fixed time, reducing the available space up to 67% for 45 min. This resulted in an increase of fish stocking density from 16 kg/m^3^ to 48 kg/m^3^. After that, the PVC structure was folded again, maintained in the 3000 L tank for 5 additional min, and then removed, enabling the fish to resume free swimming (**Figure 1B**). The AEFishBIT devices were programmed to record physical activity and respiratory frequency for 2 minutes every 5 minutes throughout the entire protocol, encompassing one hour previous to the stress, the 65−minute stress test, and the subsequent 4−hour recovery phase. The device operated at a sampling frequency of 100 Hz, and the software pre-processing of raw data was made as described elsewhere (Martos-Sitcha et al., 2019; Ferrer et al., 2020). At the conclusion of the monitoring period, all the AEFishBIT devices were successfully retrieved and pre-processed data were downloaded for tracking the recorded behavioral traits.

### 2.7 Statistical analyses

Statistically significant differences (p < 0.05) on growth performance, blood biomarkers, gene expression, and behavioural stress response were assessed by one- and two-way ANOVA followed by a Holm-Sidak *post hoc* test, using the SigmaPlot software 14.5 (Systat Software, San Jose, CA, USA). Potential tank effects were evaluated separately for each sampling time point in order to avoid confounding temporal variation with dietary effects. Growth performance parameters, including initial body weight (IBW), final body weight (FBW), feed conversion ratio (FCR), specific growth rate (SGR), feed intake, and related biometric indices, were analysed using tank means, considering each tank as the true experimental unit to avoid pseudoreplication. In contrast, blood biomarkers and gene expression data were analysed at the individual level using linear mixed-effects models implemented with the lme4 and lmerTest packages in R. For each variable, diet was included as a fixed effect while tank identity nested within diet was incorporated as a random effect. Tank-related variance components were extracted from the variance–covariance structure of the models, and the relative contribution of tank variability was quantified through the intraclass correlation coefficient (ICC), calculated as the ratio between tank variance and total residual variance. For the analysis of the histological scoring, the non-parametric Kruskal-Wallis test, followed by Dunn’s post-test for multiple comparisons was applied. Gene expression patterns were further analysed by partial least-squares discriminant analysis (PLS-DA) using EZinfo v3.0 (Umetrics, Umeå, Sweden). Unsupervised exploration of gene expression profiles was performed using Principal Component Analysis (PCA) in R (version 4.4.0). Prior to analysis, variables with zero variance were removed. PCA was computed using scaled data (unit variance scaling) to ensure comparability across features. Sample clustering patterns were visualised based on the first two principal components, and the proportion of explained variance was reported for each axis. Confidence ellipses representing the multivariate dispersion of each dietary group were calculated using a multivariate t-distribution with a 95% confidence level. To further assess differences among groups, supervised discrimination between dietary groups was assessed using Partial Least Squares Discriminant Analysis (PLS-DA) using the ropls package in R. Model robustness, predictive ability and potential overfitting were evaluated through repeated permutation testing (n = 500 permutations) and 7-fold cross-validation was performed using the Bioconductor R package ropls (Thévenot et al., 2015). Model quality was assessed using R2Y (cum) and Q2 (cum) metrics, together with score plots, loading plots, and outlier detection. The list of genes contributing to group separation was determined by the minimum Variable Importance in the Projection (VIP) values. Discriminant genes were considered with a VIP threshold ≥ 1.0 (Li et al., 2012; Kieffer et al., 2016).

## 3 Results

### 3.1 Comparative growth performance among dietary treatments

As shown in **Table 4**, fish fed the experimental diets exhibited a high feed acceptance throughout the feeding trial, growing from an initial average body weight of 13 g in May 2022 to 355–360 g by February 2023. No significant differences were observed in any of the analysed growth parameters at T_1_ and T_2_ sampling times. However, by the end of the trial, some differences in body length and Fulton’s condition factor (CFK) were noted between fish fed the CTRL diet and those fed the PAP and ALT experimental diets. These differences, though statistically significant, were almost negligible (less than 1.5%), and likely attributable to the low experimental variability of replicated tanks. Indeed, all dietary groups exhibited accelerated growth with an overall specific growth rate (SGR) of 1.24-1.25, but this rate progressively declined over the trial, dropping from 3 in early summer to 1.3 in autumn, and nearly halting in winter (SGR = 0.18-0.19). Likewise, the overall feed conversion ratio (FCR) was 1.23-1.24 across dietary treatments, starting with values below 1 during the first two months of the trial and increasing to 1.6-1.7 during overwintering. Other biometric parameters, such as muscle fat content, HSI and VSI, also remained nearly unchanged among dietary treatments, although a clear time effect was observed for both HSI and muscle fat content. Specifically, HSI increased from 1.7-2% in November to 2.2-2.4% in February, while muscle fat content declined from 10-11.3% to 8.5-10% over the same period (**Supplementary Table 4**).

**Table 4:** Data on growth performance of gilthead sea bream fed the CTRL and experimental diets (PAP, ALT) from May-2022 to February-2023.

	CTRL	PAP	ALT	*P ^1^*
Period 1 (day 0 - day 55)				
Initial Body Weight (g)	13.18±0.04	13.14±0.05	13.09±0.09	0.639
Final Body Weight (g)	70.47±0.14	70.17±0.17	70.38±0.06	0.360
Final Body Length (cm)	13.71±0.26	13.92±0.29	13.97±0.34	0.075
Final CFK ^2^	2.59±0.01	2.57±0.01	2.57±0.01	0.385
Feed Intake (g DM/fish)	49.43±0.08	49.17±0.11	50.19±0.32	0.072
Relative Weight Gain (%)	434.80±1.06	432.86±1.31	437.53±0.10	0.092
SGR (%) ^3^	3.11±0.01	3.10±0.01	3.11±0.00	0.164
FCR ^4^	0.87±0.01	0.87±0.01	0.88±0.01	0.385
Period 2 (day 55 - day 164)				
Initial Body Weight (g)	70.47±0.14	70.17±0.17	70.38±0.06	0.360
Final Body Weight (g)	296.42±0.49	293.50±1.57	297.92±1.34	0.170
Final Body Length (cm)	22.44±0.17	22.17±0.09	22.01±0.04	0.156
Final CFK ^2^	2.63±0.07	2.69±0.05	2.80±0.01	0.176
Liver Weight (g)	5.15±0.27	5.39±0.34	5.92±0.38	0.257
Viscera Weight (g)	19.67±0.92	18.44±0.83	19.78±0.85	0.491
Feed Intake (g DM/fish)	49.43±0.08	49.17±0.11	50.19±0.32	0.072
HSI (%)	1.70±0.08	1.90±0.09	1.99±0.11	0.095
VSI (%)	6.52±0.27	6.53±0.19	6.47±0.18	0.980
Muscle Fat (%)	10.08±0.46	11.30±0.54	11.37±0.65	0.196
Relative Weight Gain (%)	320.70±1.51	318.30±3.25	323.27±1.90	0.430
SGR (%) ^3^	1.32±0.01	1.32±0.01	1.33±0.01	0.385
FCR ^4^	1.13±0.01	1.13±0.01	1.13±0.00	0.829
Period 3 (day 164 - day 266)				
Initial Body Weight (g)	296.42±0.49	293.50±1.57	297.92±1.34	0.170
Final Body Weight (g)	360.42±0.58	355.25±1.59	356.71±0.70	0.083
Final Body Length (cm)	23.47±0.03^a^	23.17±0.08^b^	23.12±0.05^b^	**0.033**
Final CFK ^2^	2.79±0.01^b^	2.85±0.01^a^	2.89±0.03^a^	**0.045**
Liver Weight (g)	7.99±0.53	7.72±0.50	8.28±0.39	0.613
Viscera Weight (g)	22.96±0.95	22.85±1.18	22.66±0.74	0.975
Feed Intake (g DM/fish)	102.34±0.66	100.67±0.82	99.45±1.45	0.283
HSI (%)	2.17±0.12	2.26±0.10	2.34±0.09	0.482
VSI (%)	6.44±0.18	6.84±0.17	6.64±0.18	0.305
Muscle Fat (%)	8.49±0.58	10.00±0.54	9.25±0.59	0.187
Relative Weight Gain (%)	21.65±0.19	20.81±0.34	19.66±0.62	0.097
SGR (%) ^3^	0.19±0.00	0.19±0.01	0.18±0.01	0.164
FCR ^4^	1.60±0.03	1.65±0.03	1.7±0.03	0.142

^1^ One-way ANOVA *P-value.*

^2^ Fulton’s body condition factor, CFK = 100 x (body weight/standard length^3^).

^3^ Specific growth rate, SGR = 100 x (ln final body weight – ln initial body weight)/days.

^4^ Feed conversion ratio, FCR = 100 × (dry feed intake/wet weight gain). Values are the mean ± SEM of duplicated tanks. P-values of One-way ANOVA are shown. Different letters indicate statistically significant differences among groups (Holm-Sidak *post hoc* test, *P* < 0.05).

### 3.2 Growth performance in a context of climate change

**Figure 2:**
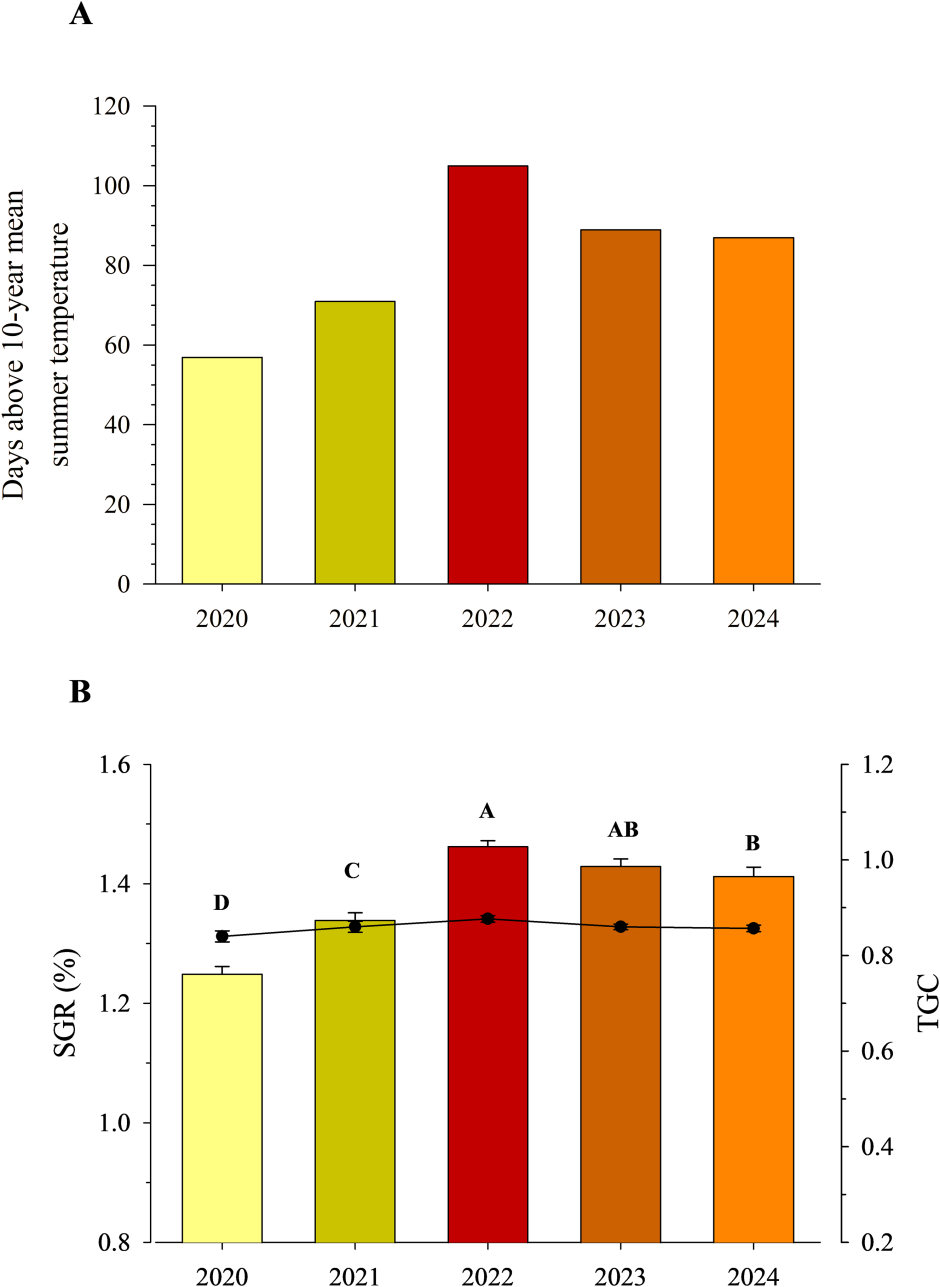
**(A)** Number of summer days in each year that exceeded the 10-year mean summer temperature, illustrating interannual variability in thermal conditions. **(B)** Growth performance of consecutive stocks. Bars represent the specific growth rate (SGR; % day⁻¹) of fish reared in each year, expressed as the mean ± SEM of duplicate tanks and calculated up to the commercial harvest size (300–350 g). Different letters indicate significant differences between the growth performance (SGR) of stocks reared across different years. Connected dots represent the thermal growth coefficient (TGC) for each stock; the vertical error bars associated with the dots indicate the SEM of TGC calculated from duplicate tanks. TGC was calculated as: TGC=((Wf^1/3^−Wi^1/3^)/∑(T×Δt)) ×1000, where Wi and Wf are the initial and final body weights (g), T is the mean water temperature (°C), and Δt is the number of days in each temperature interval.

Long-term temperature records from the IATS facilities (2013-2023) reveal an overall warming trend of 0.1 °C per year, which intensified to 0.2 °C per year during the summer months (June-September). Thus, over the last ten years, mean annual and summer SSTs averaged 18.98 °C and 24.98 °C, respectively. Notably, 2022, 2023, and 2024 showed pronounced thermal anomalies, with mean summer temperatures exceeding 26 °C and peak values surpassing 30 °C. In consequence, the number of days above the decadal summer mean was higher in 2022, 2023 and 2024 than in previous years (**Figure 2A**), indicating increased thermal availability. In line with this, growth up to harvest (300–350 g) differed among production cycles (**Figure 2B**), with overall SGR values ranging from 1.25 to 1.45. The 2022 stock exhibited the faster growth, followed by 2023 and 2024, being all of which significantly higher than in 2020 and 2021, while TGC remained almost equal across all the recorded production cycles (0.84-0.88). Together, these observations suggest that elevated temperatures primarily drove accelerated growth, while overall growth efficiency per degree-day remained almost constant with FCR values of 1.1-1.2.

### 3.3 Dynamics of histopathological scoring

**Figure 3:**
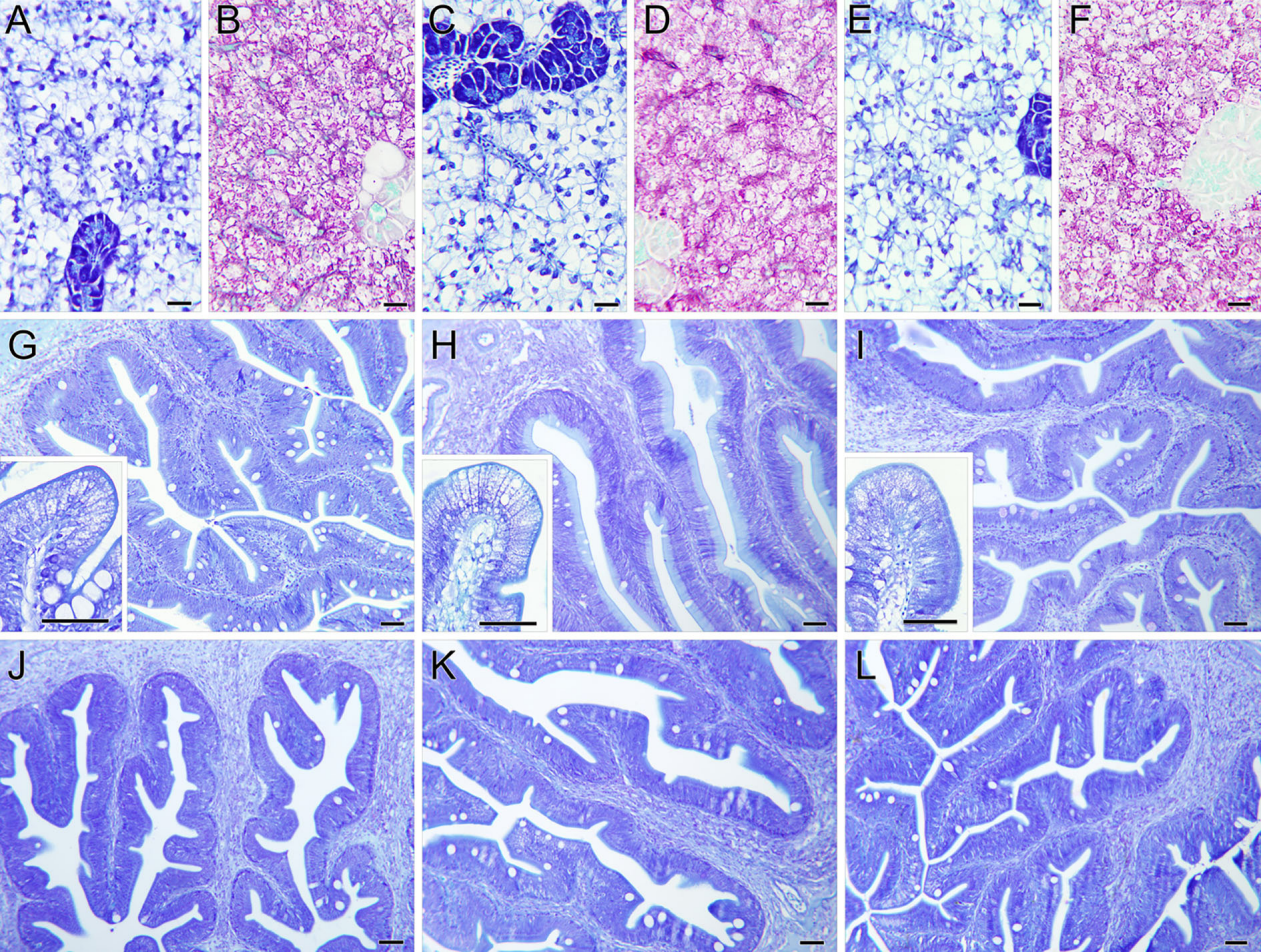
Liver and intestine histology at the last sampling point (t_3_, February 2023) of fish fed the CTRL diet **(A, B, G, J)**, the PAP diet **(C, D, H, K)** and the ALT diet **(E, F, I, L)**. Liver **(A–F)** sections present no histopathological signs and no differences among fish fed the experimental diets. The anterior **(G–I)** and the posterior **(J–L)** intestinal segments present no histopathological signs and no differences are observed among fish fed the experimental diets. Giemsa staining **(A, C, E, G–L)** and PAS staining **(B, D, F)**. Scale bars = 50 µm in intestinal sections, and = 20 µm in liver sections.

At the end of the 2022 feeding trial (February 2023, T_3_), no remarkable histopathological alterations were observed in the liver of CTRL and experimental groups (**Figures 3A–F**). A moderate degree of glycogen and lipid deposition was present in hepatocytes. In the AI, inflammatory markers were detected at low levels, and only slight lipid vacuolization of enterocytes at the tips of the intestinal folds was observed in all the dietary treatments (**Figures 3G–I**). The PI showed no signs of inflammation (**Figures 3J–L**), though low goblet cell numbers were noted in all dietary groups. Temporal changes in histopathological scoring from the beginning to the completion of the trial are provided as **Supplementary Material**. Briefly, a significant increase in lipid storage within hepatocytes was observed over time in both the CTRL and experimental dietary groups from July onward (**Supplementary Figures 2A, B**), which was accompanied by a decrease in glycogen depots in fish fed the ALT diet (**Supplementary Figure 2C**). In the AI, goblet cell abundance showed an increasing trend from July onward in the CTRL group, while in the other experimental groups the highest values were registered in July and February (**Supplementary Figures 3A**, **B**). A slight increase in lipid vacuolization of enterocytes was also noted over time in the AI across all dietary groups (**Supplementary Figure 3C**), though no inflammatory markers were prominent. In contrast, in the PI, goblet cell numbers decreased similarly in all three experimental groups from November 2022 to February 2023, accompanied by the onset of mild submucosal hyperplasia (**Supplementary Figure 4A–C**).

### 3.4 Dynamics of blood biochemical markers among dietary groups

**Figure 4:**
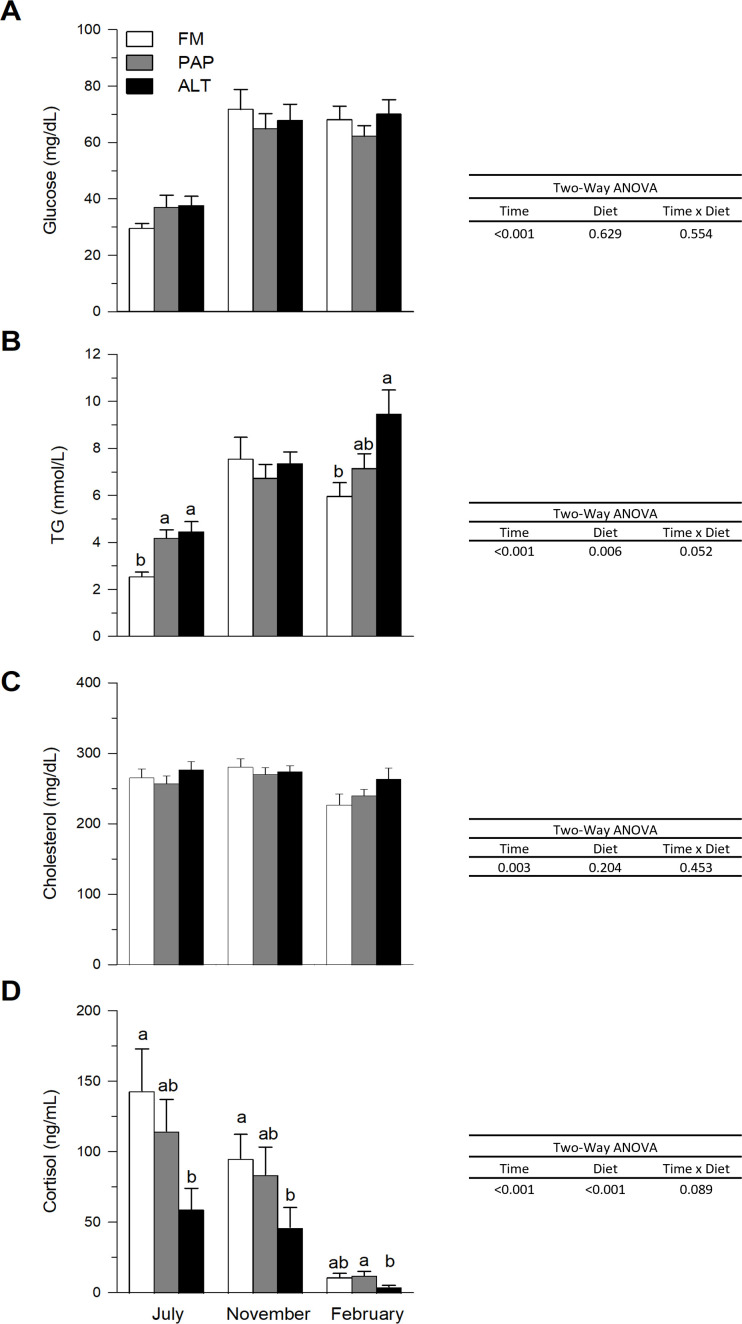
Effect of diet on blood biochemistry at three sampling periods. Plasma levels of glucose **(A)**, triglycerides **(B)**, cholesterol **(C)** and cortisol **(D)** in each sampling time. Different letters indicate statistically significant differences (Holm-Sidak *post hoc* test, *p* < 0.05) between experimental groups in each sampling time. Two-way ANOVA p-values results are shown at the right side of each figure.

A pronounced time effect was evidenced over the trial for circulating glucose and TG levels, accompanied by marked reductions in cortisol and, to a lesser extent, cholesterol concentrations from the warmer month of July to the colder month of February (**Figure 4**). A diet effect was not found on plasma glucose and cholesterol levels (**Figures 4A, C**). In contrast, TG and cortisol levels exhibited a marked diet effect displaying opposite trends (**Figures 4B, D**). According to which, the highest TG concentration was achieved by ALT fish in winter, while the same fish shaped reduced cortisol concentration across all the trial with the lowest values at the last sampling point in February. Tank-effect assessment further indicated that the contribution of tank-associated variability was generally low for all the measured markers.

### 3.5 Tissue-specific gene expression profiling among dietary groups

**Figure 5:**
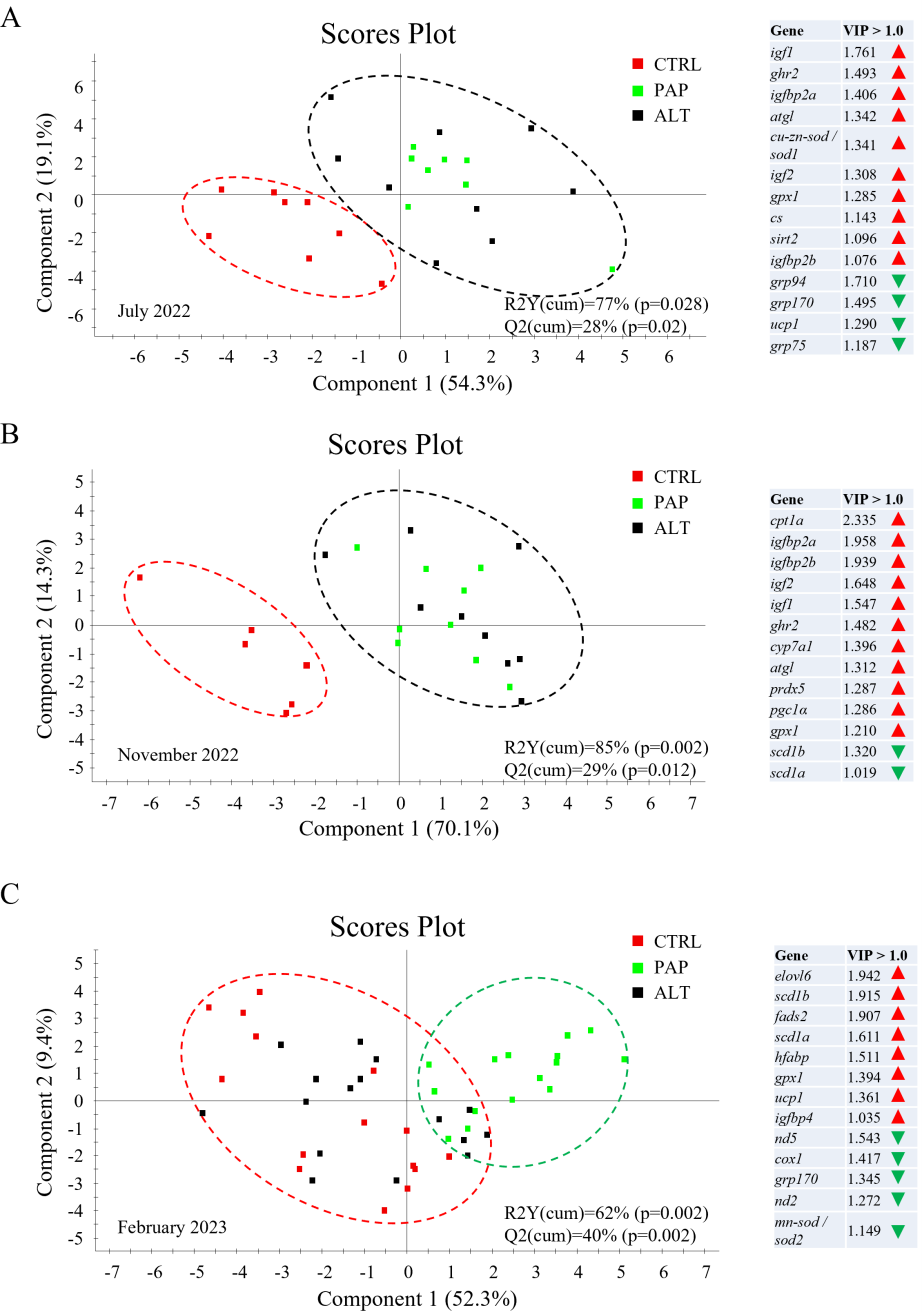
Two-dimensional PLS-DA score plot of liver gene expression at sampling period t_1_ **(A)**, t_2_ **(B)** and t_3_ **(C)**, representing the distribution of the samples between the first two components in the model. The cumulative explained R2Y(cum) and predicted Q2(cum) variance, as well as the p-values of the permutation plot can be found at the bottom-right side of the figure. Discriminant genes are ordered by variable importance in the projection (VIP). Red and green VIP values indicate up- and down-regulation, respectively, in the divergent group in comparison to the grouped experimental diets.

**Figure 6:**
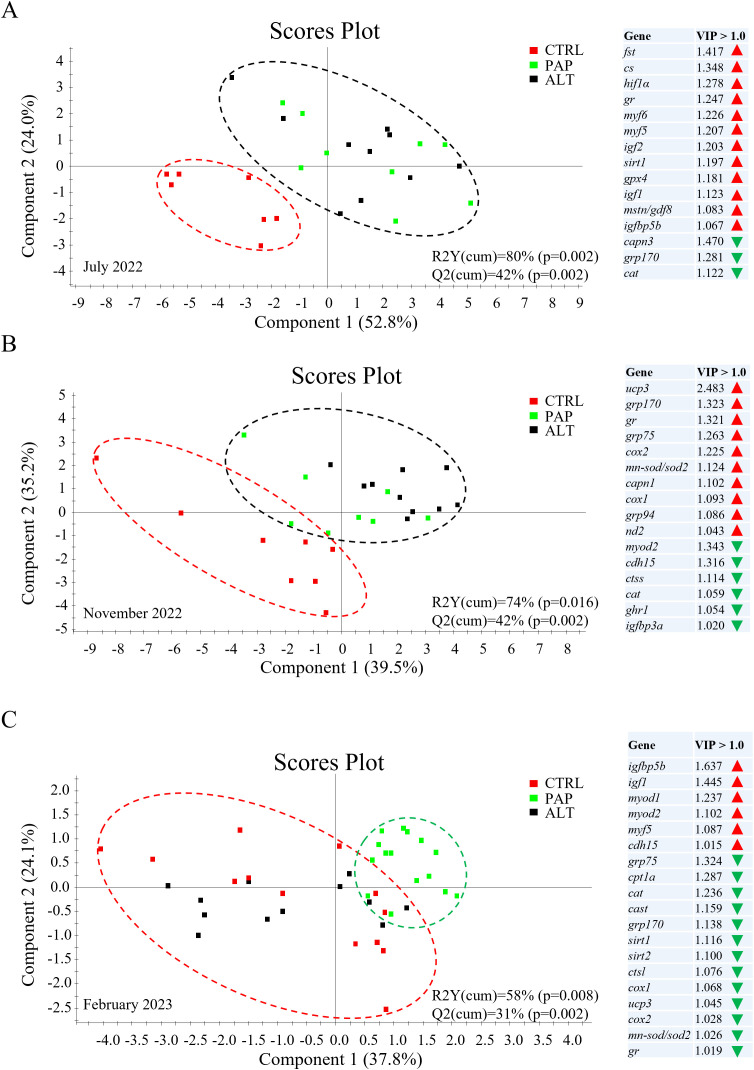
Two-dimensional PLS-DA score plot of white skeletal muscle gene expression at sampling period t_1_ **(A)**, t_2_ **(B)** and t_3_ **(C)**, representing the distribution of the samples between the first two components in the model. The cumulative explained R2Y(cum) and predicted Q2(cum) variance, as well as the p-values of the permutation plot can be found at the bottom-right side of the figure. Discriminant genes are ordered by variable importance in the projection (VIP). Red and green VIP values indicate up- and down-regulation, respectively, in the divergent group in comparison to the grouped experimental diets.

Gene expression profiling in response to dietary treatment evolved in a tissue-specific manner over time, highlighted by unsupervised PCA analyses, which showed a gradual increase in the separation among CTRL and experimental dietary groups over time. In particular, sample clustering at the final sampling point (T_3_) (**Supplementary Figures 5A–C**) appeared more distinct than at earlier stages, although partial grouping patterns could already be present at T_1_ and T_2_. Based on these exploratory results, supervised PLS-DA models were subsequently constructed and validated (**Supplementary Figure 6**) to further characterize diet-associated transcriptional differences. PLS-DA analyses confirmed that the strongest discriminatory patterns occurred at the final sampling point, where it was observed a relatively high number of differentially expressed genes, that varied from 21 in liver and 29 in WSM to 35 in AI (**Supplementary Tables 5–7**). Nevertheless, despite a lower degree of separation, early sampling points also showed emerging clustering trends. In fact, while early transcriptional shifts likely reflect the initial and different adaptative features to each CTRL and experimental diet, the final sampling time would amplify the cumulative and divergent effects of dietary exposure. Thus, for liver and to a lesser extent in WSM, the results suggested a separation between CTRL fish and PAP-ALT groups at T_1_ and T_2_ (**Figures 5A, B**, **6A, B**), while at T_3_, the most pronounced divergence was observed with PAP fish that clustered separately from the CTR-ALT counterpart (**Figures 5C**, **6C**), which was driven by 13 and 19 discriminant genes (VIP > 1.0) in liver and WSM, respectively. In liver, such PAP-specific transcriptional signatures were shared by the up-regulation of lipogenic enzymes (fatty acid desaturase 2, *fads2*; elongation of very long-chain fatty acids protein 6, *elovl6*; and stearoyl-CoA desaturases, *scd1a*, *scd1b*), heart-type fatty acid-binding protein (*hfabp*), uncoupling protein 1 (*ucp1*), antioxidant enzymes (glutathione peroxidase 1, *gpx1*) and binding proteins of insulin-like growth factor (*igfbp4*). In contrast, the expression of mitochondrial respiratory enzyme subunits of NADH dehydrogenase (*nd2*, *nd5*) and cytochrome c oxidase subunit 1 (*cox1*), in combination with the mitochondrial superoxide dismutase (*mn-sod/sod2*), and the 170 kDa glucose regulated stress protein (*grp170*) were down-regulated, explaining the PLS-DA model 62% and 40% of the observed and predicted variance, respectively (**Figure 5C**). In WSM, the separated clustering of PAP fish was driven by the down-regulated expression of markers of fatty acid oxidation, mitochondria respiration and uncoupling, energy sensing and protein turnover. In particular, PAP fish exhibited a reduced expression of carnitine palmitoyltransferase 1a (*cpt1a*), uncoupling protein 3 (*ucp3*), cytochrome c oxidase subunits (*cox1*, *cox2*), sirtuin 1 (*sirt1*) and sirtuin 2 (*sirt2*), together with a diminished expression of antioxidant stress markers (*mn-sod/sod2*; *grp170*; glucose-regulated protein 75 kDa (*grp75*), catalase (*cat*); glutathione reductase, *gr*), and proteolytic-related enzymes such as calpastatin (*cast*) and cathepsin L (*ctsl*). Conversely, insulin-like growth factor 1 (*igf1*), insulin-like growth factor binding protein 5b (*igfbp5b*), cadherin 15 (*cdh15*), and several myogenic regulatory factors (*myod1*, *myod2*, *myf5*) were up-regulated in PAP fish. Collectively, these discriminant genes explained approximately 58% of the observed variance and 31% of the predicted variance (**Figure 6C**).

**Figure 7:**
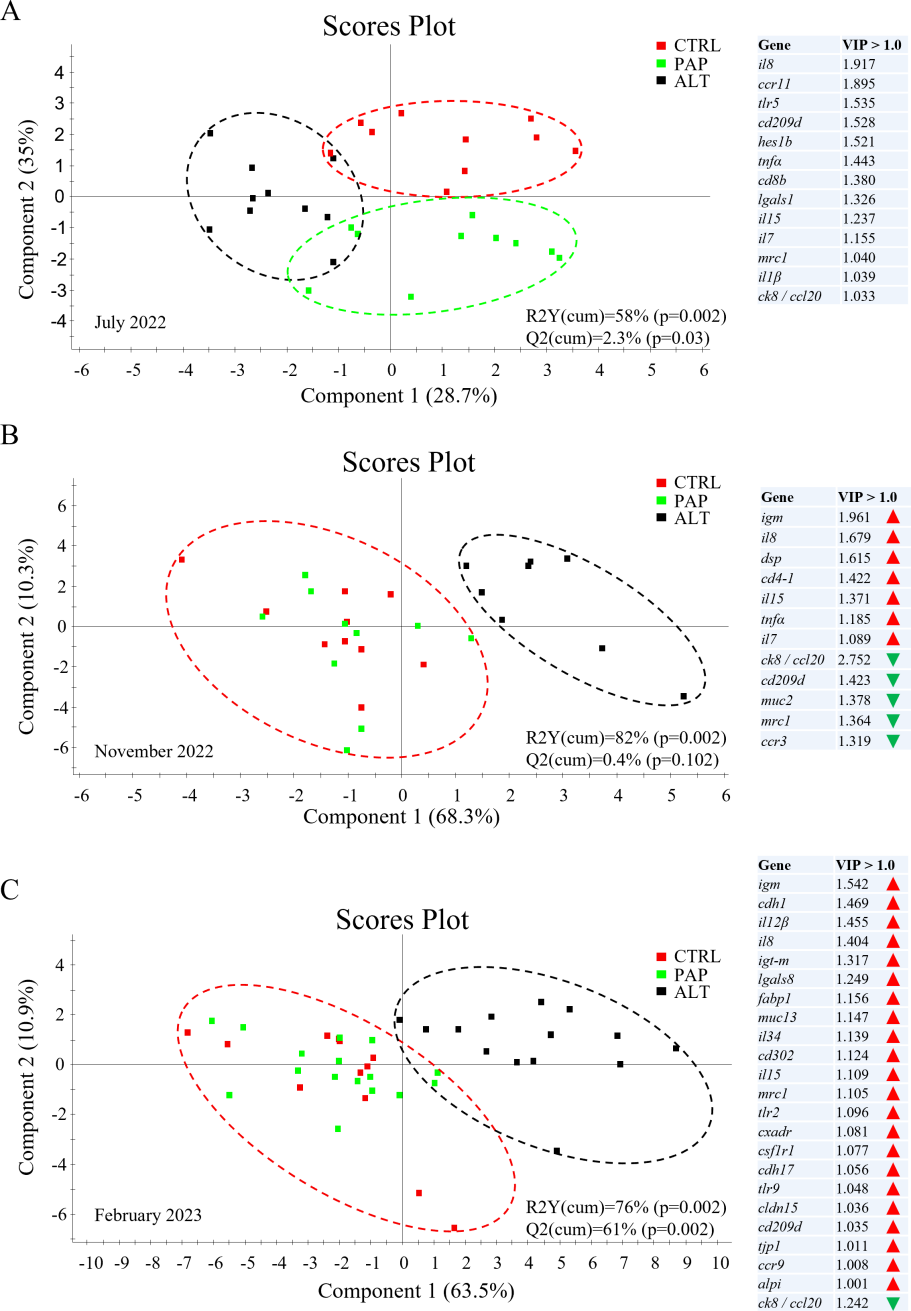
Two-dimensional PLS-DA score plot of anterior intestine gene expression at sampling period t_1_ **(A)**, t_2_ **(B)** and t_3_ **(C)**, representing the distribution of the samples between the first two components in the model. The cumulative explained R2Y(cum) and predicted Q2(cum) variance, as well as the p-values of the permutation plot can be found at the bottom-right side of the figure. Discriminant genes are ordered by variable importance in the projection (VIP). Red and green VIP values indicate up- and down-regulation, respectively, in the divergent group in comparison to the grouped experimental diets.

In the AI, the three dietary groups resulted separated at T_1_ (**Figure 7A**), while at T_2_ and T_3_ (**Figures 7B, C**), ALT instead of PAP fish maintained a distinct transcriptional profile in comparison to the clustered CTRL-PAP group. This separation was initially driven by 12 discriminant genes, which increase up to 23 at the last sampling time with a higher percentage of both observed (76%) and predicted variance (61%). Such separation resulted in a consistent up-regulation in the ALT group of a broad number of markers of epithelial barrier integrity (claudin 15, *cldn15*; cadherin 1, *cdh1*; cadherin 17, *cdh17*; tight junction protein 1, *tjp1*; coxsackievirus and adenovirus receptor, *cxadr*; transcriptional factor HES-1B, *hes1b*), nutrient transport (fatty acid-binding protein 1, *fabp1*; intestinal alkaline phosphatase, *alpi*), mucus production (mucin 13; *muc13*), and immune activation through changes in the expression pattern of cytokines (interleukin 8, *il8*; interleukin 12 subunit beta, *il12β*; interleukin 15, *il15*; interleukin 34, *il34*), chemokine receptors (C-C chemokine receptor type 9, *ccr9*; C-C chemokine receptor type 11, *ccr11*), pattern recognition receptors (galectin 8, *lgals8*; toll-like receptor 2, *tlr2*; toll-like receptor 9, *tlr9*; CD209 antigen-like protein D, *cd209d*, CD302 antigen, *cd302*), macrophage markers (macrophage colony-stimulating factor 1 receptor 1, *csf1r1*; cluster of differentiation 4, *cd4-1*; macrophage mannose receptor 1, *mrc1*), and immunoglobulins (immunoglobulin M, *igm*; immunoglobulin T membrane-bound form, *igt-m*). The only exception is C-C motif chemokine 20 (*ck8/ccl20*), that was down-regulated in ALT fish in comparison to PAP group and to a lower extent in relation to CTRL fish. For all these genes, considering their expression in different tissues, mixed-model analyses indicated that the variance attributable to tank was minimal, supporting that the observed differences were primarily associated with dietary influence.

### 3.7 Confinement stress-test response

**Figure 8:**
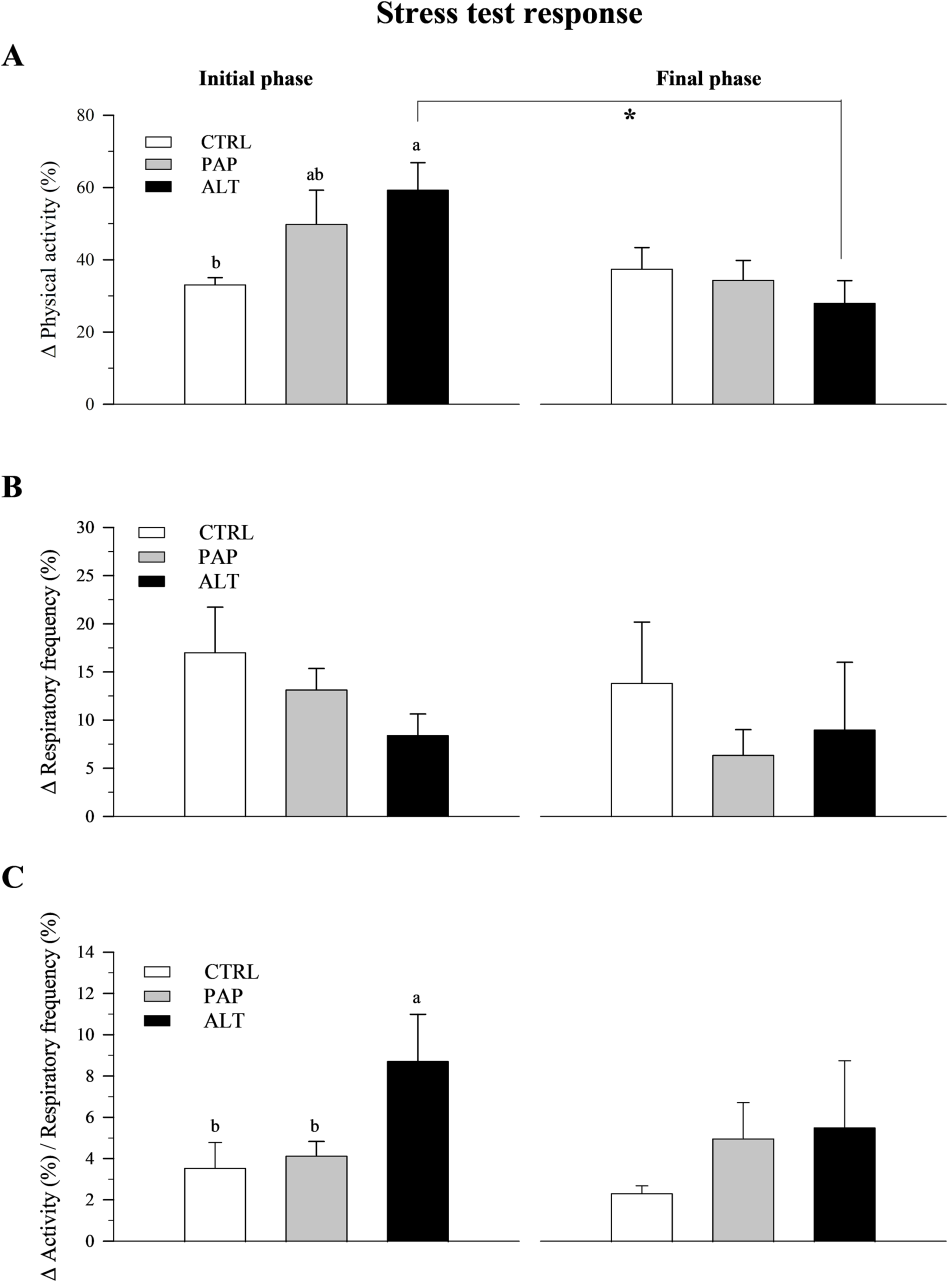
Stress test response of each experimental group shown as the increase of physical activity **(A)**, increase of respiratory frequency **(B)** and the ratio increase of physical activity/respiratory frequency **(C)**. Physical activity and respiratory frequency increases were calculated taking as reference the highest registered value of the initial and final phase and the mean basal level (1-hour before the test). Different letters indicate statistically significant differences (Holm-Sidak *post hoc* test, *p* < 0.05) between experimental groups. *, indicates significant differences between the initial and final phase.

Fish from all three experimental groups displayed two distinct peaks during the confinement test: an early response at the onset of confinement and a late response after approximately 15–20 minutes. These patterns were observed for both physical activity (**Supplementary Figures 7A–C**) and respiratory frequency (**Supplementary Figures 7D–F**). During the early phase, fish in the ALT group showed a significantly greater increase in physical activity than those in the CTRL group, while the PAP group exhibited an intermediate response (**Figure 8A**). In contrast, no significant differences in physical activity were detected among groups during the late phase, although a trend was observed in which activity levels were highest in the CTRL group, followed by PAP and ALT. Respiratory frequency did not differ significantly among groups or between the early and late phases (**Figure 8B**). However, a trend was evident during the early phase, with respiratory frequency increasing in the order CTRL > PAP > ALT. Consequently, the activity-to-respiratory frequency ratio during the early phase was significantly higher in the ALT group compared with the other groups (**Figure 8C**), with a similar but non-significant trend observed during the late phase.

## 4 Discussion

Prolonged exposure to elevated temperatures can compromise fish physiology, welfare, and overall productivity (Cascarano et al., 2021). However, analysis of growth records across five consecutive production cycles (2020-2025) revealed that, under our experimental conditions, the 2022 cohort exhibited the fastest growth (**Figure 2**). This coincided with the warmest summer in the 2013–2025 series, a scenario representative of future conditions in the Western Mediterranean (Haberle et al., 2024). Within this context, and in light of the need to develop adaptive nutritional strategies to cope with climate-driven fluctuations, the present study evaluated the performance of gilthead sea bream reared with three experimental diets differing in dietary lipid and protein sources. Notably, growth performance remained high across all dietary treatments under an unprecedented thermal regime. However, each dietary treatment caused tissue-specific differences in gene expression (liver, WSM, AI) and behavioral responses, which should be considered in the future to ensure the production of robust, efficient and fast-growing fish at the farm level in the context of global warming.

Under the warm thermal conditions recorded in 2022, a closer look revealed that the growth performance of fish fed the PAP and ALT diets was comparable to that of the CTRL group, with overall SGR and FCR values falling within the optimal range reported for gilthead sea bream of this size class (Simó-Mirabet et al., 2018; Šegvić-Bubić et al., 2025). Likewise, no differences among dietary groups were observed in biometric indices such as HSI and VSI, while histopathological analyses highlighted the preservation of hepatic and intestinal architecture without any clear evidence of inflammation or tissue damage in either the liver or AI over the trial (**Figure 3**). These results align with previous studies showing satisfactory growth performance and overall good health in gilthead sea bream fed diets enriched with processed animal proteins (PAPs), insect meals, or microbial biomass (Di Rosa et al., 2023; Donadelli et al., 2024; Gai et al., 2023; Marchi et al., 2023; Sarmiento et al., 2025). At the same time, they contrast with earlier reports suggesting that prolonged exposure to temperatures above 28 °C can lead to growth impairment, inflammatory responses, and oxidative stress in this species (Madeira et al., 2014; Zarantoniello et al., 2021). In the present study, however, the lack of detectable growth penalties suggests the maintenance of a balanced allostatic load throughout the experimental period, likely supported by species-specific adaptive remodeling over time of multiple physiological and metabolic processes. Specifically, WSM fat content gradually declined from approximately 11-10% in November to 10-8.5% in February, while HSI increased over the same period from 1.7-2.0% to 2.2-2.4%. These trends were supported by histopathological observations, which showed a moderate increase in hepatic lipid depots during winter, along with mild lipid vacuolization in enterocytes. Taken together, these findings indicate a redistribution of tissue energy reserves, with the liver acting as a metabolic buffer by dynamically regulating lipid synthesis, storage, and oxidation in response to changing biotic and abiotic conditions (Rui, 2014; Topić Popović et al., 2023). Such metabolic flexibility likely contributes to the maintenance of somatic growth and physiological homeostasis under sustained warm and cold adaptation processes, while potentially enhancing resilience to additional environmental pressures, including pollutants, microplastics, and other anthropogenic contaminants increasingly present in coastal farming systems (Fred-Ahmadu et al., 2024; Matias et al., 2025). Under these conditions, progressive warming of the Western Mediterranean may enhance metabolic efficiency and shorten production cycles by 1–2 months to reach a harvest size of 300–350 g, although production would operate close to the upper thermal tolerance limit of the species (Kir, 2020; Haberle et al., 2024).

At the systemic level, seasonal shifts in energy allocation in gilthead sea bream were also reflected in circulating metabolites (**Figure 4**). Thus, both circulating glucose and TG concentrations increased from July to February, indicating increased energy mobilization as fish approached the colder months. Besides, while glucose levels were similar across dietary groups, TGs exhibited a diet-dependent effect with ALT-fed fish showing the highest concentration by February. This seasonal TG dynamics align with the observations of Vargas-Chacoff et al. (2009), who reported low levels in summer with progressive increases into autumn and winter. In contrast, circulating glucose showed an opposite seasonal trend in their pond-reared fish, peaking in summer and declining toward winter. This discrepancy likely reflects differences in rearing conditions, growth stages, or feeding regimes, with glucose responding rapidly to environmental and nutritional changes, while TGs provide a more stable measure of seasonal energy storage (Feidantsis et al., 2018). Otherwise, it is noteworthy that plasma cortisol levels were consistently lower across all the trial in ALT-fed fish in comparison to both CTRL and PAP fish. Previous studies in gilthead sea bream have reported minimal or non-significant changes in cortisol levels following the dietary inclusion of insect meal (Gai et al., 2023; Basto et al., 2024), but intriguingly a slight downward trend has also been observed with the use of black soldier fly as an alternative feed ingredient in fish feeds (Di Rosa et al., 2023). Certainly, most nutritionally mediated effects on baseline stress markers are generally modest, but they can be reinforced through the synergistic action of other functional feed additives. Although it remains unclear whether such synergism occurred in the present study, it is worth noting that porcine blood hydrolysate (PBSH), enriched in multifunctional bioactive peptides, has shown antioxidant, hypoglycemic and anti−inflammatory activities (Moreno-Mariscal et al., 2025a). Moreover, in gilthead sea bream, supplementation of ALT diet with PBSH was reported to lower basal cortisol, reduce aggression, decrease resting metabolic rate, enhance swimming performance (a 20% increase in critical swimming speed) and shift the intestinal microbiota toward increased representation of abundant genera such as *Aureimonas* and *Halomonas* (Moreno-Mariscal et al., 2025b).

Overall, the above findings indicate that ALT-based diet formulations can influence energy metabolism and basal Hypothalamus-Pituitary-Interrenal (HPI) axis activity, potentially enhancing resilience to environmental or handling stress. Indeed, nutritional background clearly influenced behavioral and activity responses during a challenge test under reduced space availability (**Figure 8**). Measurements obtained with the AEFishBIT biologger showed that, at the onset of the challenge, ALT-fed fish displayed a higher activity-to-respiratory-frequency ratio, indicating a more proactive coping style during the early phase of the response. Among farmed fish, proactive individuals characteristically combine elevated locomotor activity with lower ventilatory reactivity and reduced cortisol responsiveness to aquaculture stressors (Vindas et al., 2018; Castanheira et al., 2017), a profile commonly associated with dampened HPI-axis reactivity and monoaminergic modulation (Vindas et al., 2018; Backström and Winberg, 2017; Höglund et al., 2020). Thus, in the present study, the rapid onset of the elevated activity-to-respiratory-frequency ratio observed in ALT-fed fish likely reflects a combination of low pre-existing cortisol tone, rapid autonomic and neurotransmitter regulation, and metabolic preparedness, rather than a newly established HPI-axis set point, where acute cortisol dynamics unfold over tens of minutes to hours (Salamanca et al., 2025). Mechanistically, diet-driven factors such as changes in tissue energy partitioning reflected by elevated plasma TG levels, or microbiota-derived metabolites originating from insect components could prime peripheral and central systems for a more active coping strategy. Specifically, insect-derived ingredients may modulate the gut-brain axis via chitin-driven microbial fermentation and short-chain fatty acid (SCFA) production, potentially affecting monoaminergic signaling and stress resilience (Rimoldi et al., 2024; Churilov et al., 2025). Direct causal links between microbiota, SCFAs, monoamine signaling, and behavior remain elusive in marine fish. Nevertheless, the present study further supports an integrated relationship between metabolic state, baseline endocrine tone, and behavioral strategy, in which the ALT diet was associated with a greater immediate activity following confinement stress.

While fish growth is often used as a primary indicator of dietary success, underlying physiological and molecular adaptations can differ substantially between dietary groups. Certainly, phenotypic evaluation in the present study showed that fish achieved optimal growth regardless of dietary treatment, without apparent signs of histopathological damage in liver and intestine tissue samples. However, gene expression analyses revealed temporal and diet-specific responses, suggesting that similar growth outcomes may be associated with compensatory physiological strategies that help maintain the metabolic homeostasis under varying nutrient conditions (Aragão et al., 2022; Fernandes et al., 2024). Indeed, a number of studies in fish have shown that diet influences oxidative load, immune responsiveness, and various biochemical markers, with effects often becoming apparent only after prolonged periods (Casu et al., 2017; Feng et al., 2025; Herrera et al., 2022; Yu et al., 2023). Accordingly, the tissue-specific gene expression patterns in the liver and WSM of CTRL and experimental groups revealed divergent transcriptional profiles that drove a clear separation of PAP-fed fish from the merged CTRL and ALT fish group at the final sampling point during the winter season (**Figures 5**, **6**). Such observation is consistent with a coordinated regulation of liver and WSM, in agreement with their central role as metabolically active tissues regulating energy substrate mobilization, synthesis and utilization (Bermejo-Nogales et al., 2015; Roques et al., 2020). Concretely, this differential nutritional regulation was driven in PAP fish by the up-regulation of hepatic lipogenic enzymes (*fads2*, *elovl6*, *scd1a/b*) alongside the down-regulation in either liver or WSM of a subset of metabolic markers, including mitochondrial fatty acid transporters (*cpt1a*), enzyme subunits of the mitochondrial respiratory chain (*nd2*, *nd5*, *cox1*, *cox2*), antioxidant enzymes (*gpx1*, *mn-sod/sod2*), molecular chaperones (*grp75*, *grp170*), and sirtuin NAD+-dependent enzymes (*sirt1*, *sirt2*) that act as crucial cellular energy sensors. Conversely, respiration uncoupling proteins were up-regulated in liver (*ucp1*) but again down-regulated in WSM (*ucp3*). Overall, this gene expression pattern is indicative of a reduced tissue metabolic activity in PAP fish, and may reflect a shift toward lipid storage of energy surplus rather than the oxidation of energy substrates for ATP production. Thus, according to the cold adaptation theory, this metabolic feature could be interpreted as a potential constraint to sustain physiological functions under cold conditions (Dao et al., 2025; White et al., 2012). In this scenario, the up-regulation in the WSM of somatotropic-related genes (*igf1*, *igfbp5b*) and fibroblast growth factors (*myf5*), in concurrence with the enhanced expression of myogenic cell differentiation factors (*myod1*, *myod2*) and cell adhesion markers (*cdh15*), may be indicative of a counter-regulatory response, similar to that achieved in European sea bass juveniles facing diets containing synthetic microplastics (viscose-rayon microfibers) (Matias et al., 2025). The metabolic adjustments observed in fish fed PAP-based diets were likely associated with differences in dietary composition, including amino acid availability, lipid classes, peptides, and other bioactive compounds (Woodgate et al., 2022). These nutritional factors may influence key nutrient-sensing pathways, such as mTOR, insulin signaling, and SREBP, thereby influencing tissue-specific metabolic regulation and overall physiological balance (Dong et al., 2017; Fernandes et al., 2024; Gong et al., 2023; Han et al., 2020; Liu et al., 2025). In this context, PAPs represent a feasible alternative to FM when inclusion levels are appropriately managed, as excessive replacement has previously been linked to negative metabolic outcomes in farmed fish (Hoerterer et al., 2022; Kumar et al., 2017). Consistent with these observations, the present study demonstrated that PAP inclusion did not impair growth performance in gilthead sea bream. Nevertheless, the detected molecular responses suggest possible long-term effects on energy substrate utilization and ATP production. Such alterations could potentially impair cold acclimation capacity during the production cycle by reducing liver and muscle metabolic activity and by promoting hepatic lipogenesis, thereby increasing the risk of steatosis.

Intestinal gene expression analyses further indicated diet-dependent modulation, with distinct temporal patterns among the experimental groups (**Figure 7**). In particular, the ALT group exhibited a different trajectory compared with the CTRL and PAP experimental groups at the final sampling point. A defining feature of the ALT formulation was the inclusion of insect meal, which may have contributed to a moderate activation of the gut immune axis, as reflected by the regulation of immune-related genes (*il8*, *il12β*, *il15*, *il34*, *tlr2*, *tlr9*, *igm*), together with markers associated with epithelial barrier integrity (*cldn15*, *cdh17*, *tjp1*). These responses are in accordance with the known immunostimulatory effects associated with insect meal components, including chitin/chito-oligosaccharides and derived chitosans, which generally activate innate immune cells and induce cytokines production through different cell surface receptors (Henry et al., 2015; Mohan et al., 2023). Apparent modulation of the mucus production was also triggered since the expression of *hes1b*, *muc2* and *muc13* was regulated by the diet. Activation of the Notch-Hes1 pathway driving intestinal epithelial cell differentiation results in absorptive enterocyte differentiation and restricts secretive goblet cell differentiation with a subsequent decrease of mucus secretion (Crosnier et al., 2005; Riera-Ferrer et al., 2024). Thus, *hes1b* upregulation would have been counterbalanced by an increased expression of the secreted mucin (*muc2*) and the membrane-bound mucin (*muc13*) involved in mucosal protection (Perez-Sanchez et al., 2013). Insect meals, particularly those derived from the black soldier fly (*Hermetia illucens*) and the yellow mealworm (*Tenebrio molitor*), have been extensively studied and shown to exert positive effects on growth performance, welfare, and antioxidant status in a wide range of species, including rainbow trout (*Oncorhynchus mykiss*), Nile tilapia (*Oreochromis niloticus*), gilthead sea bream, and European sea bass (Henry et al., 2018a, Henry et al., 2018b; Moutinho et al., 2024; Yildirim-Aksoy et al., 2020). Beyond their positive effects on growth performance, welfare, and antioxidant status, insect meals have also been reported to influence additional physiological processes. In particular, intestinal peristalsis is affected, as a 10% replacement of FM) with black soldier fly meal significantly altered both the excitatory and inhibitory components of gastrointestinal transit (Bosi et al., 2021). Moreover, insect meals play a relevant role in modulating the gut microbiota, since differences in metabolizable substrates, together with the presence of antimicrobial compounds, have been widely reported to induce shifts in intestinal microbial communities (Hirsch et al., 2019; Li et al., 2022; Stenberg et al., 2019; Terova et al., 2021; Weththasinghe et al., 2022). Therefore, all these findings support the use of insect meals as sustainable alternatives for the partial or total replacement of FM in aquafeeds (Chen et al., 2024; Gasco et al., 2020; Hua, 2021). However, such diet-induced metabolic changes may require a range of physiological adaptations by the host (Nogales-Mérida et al., 2019), including alterations in mucus secretion and the regulation of epithelial-associated factors, as observed in the present study, in the absence of any apparent histopathological damage.

**Figure 9:**
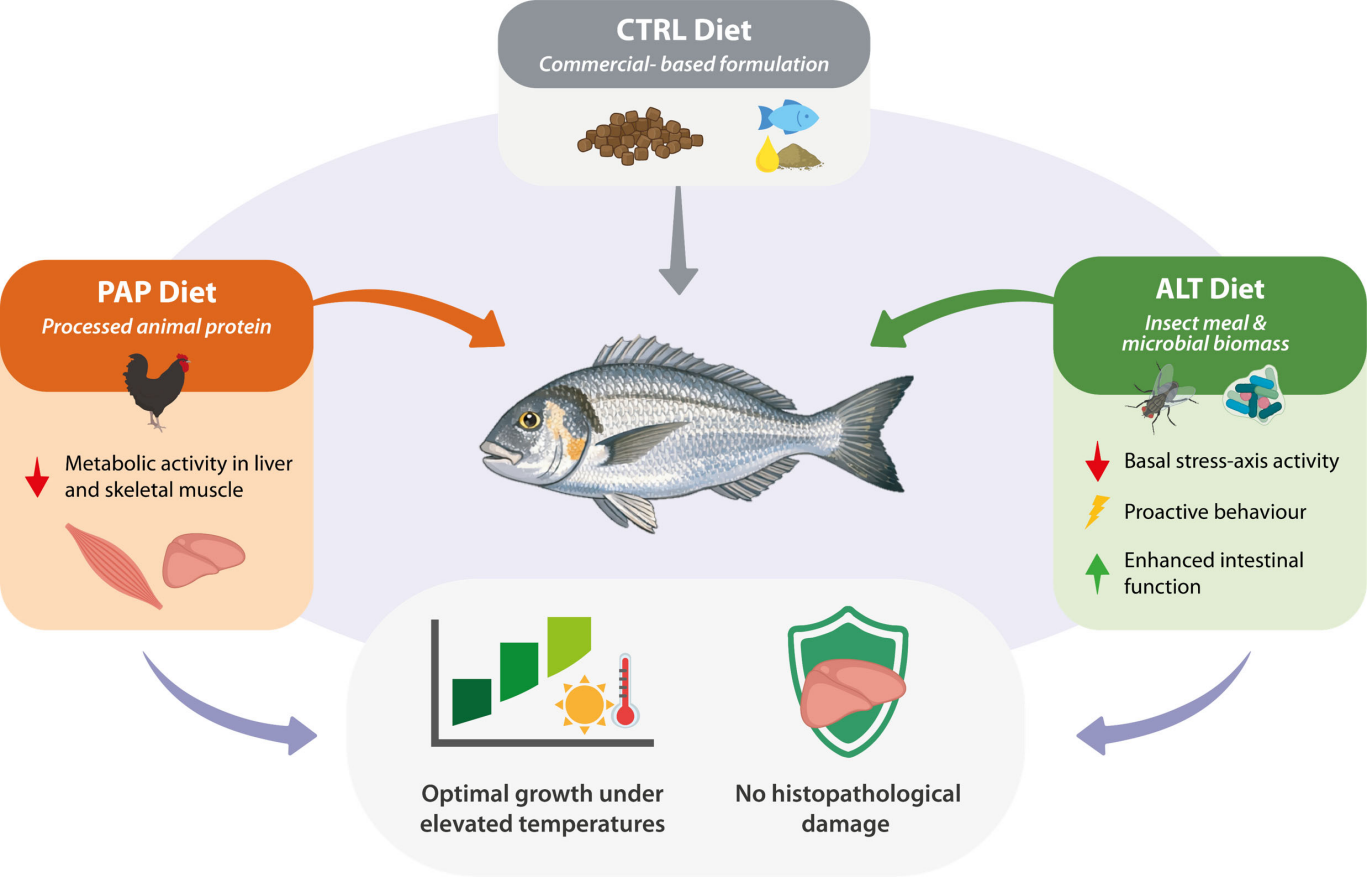
Graphical summary of the main results of fish fed the experimental diets across a production cycle. Figure created with BioRender.com.

In summary, as shown in **Figure 9**, the results demonstrate that gilthead sea bream can sustain accelerated growth under unusually warm Mediterranean summer temperatures when fed either a commercial-based diet or experimental formulations containing high levels of animal proteins, microalgal biomass, or insect proteins as alternative FM replacers. No signs of histopathological damage were observed in liver or intestinal samples, indicating preserved tissue integrity throughout the trial. However, this efficient growth was associated with distinct physiological strategies: the ALT diet was characterized by lower basal stress-axis activity, proactive behavioral responses, and enhanced intestinal transcriptional activity, suggesting a different physiological response profile to common farming stressors; while the PAP diet was associated with lower liver and WSM metabolic activity, which may represent a potential trade-off in which cold adaptation, rather than warm adaptation, could be compromised.

## Data Availability

The original contributions presented in the study are included in the article/**Supplementary Material**. Further inquiries can be directed to the corresponding author.
