# Supplementary material for: Physiologically mediated responses in gilthead sea bream (Sparus aurata) fed sustainable diets: seasonal growth under warming conditions
Source: Front Physiol. 2026 Jun 30;17:1860904. doi: 10.3389/fphys.2026.1860904 (PMC13392755; doi:10.3389/fphys.2026.1860904)
Supplement: Supplementary file 2 [file Table2.docx]

Supplementary Table 2. Primers for qPCR amplification of hepatic (*) and skeletal muscle (**^†^**) transcripts.

| **Gene** | **Symbol** | **GenBank** | **Primer** | **Amplification efficiency (%)** | |
| --- | --- | --- | --- | --- | --- |
|  |  |  |  | **Liver** | **Muscle** |
| Beta-actin | *actb****^†^** | KY388508 | F: TCC TGC GGA ATC CAT GAG A |  |  |
|  |  |  | R: GAC GTC GCA CTT CAT GAT GCT | 94.8 | 95.0 |
|  |  |  |  |  |  |
|  |  |  |  |  |  |
| elongation factor 1α | *ef1a****^†^** | AF184170 | F: CCC GCC TCT GTT GCC TTC G | 96.1 | 96.7 |
|  |  |  | R: CAG CAG TGT GGT TCC GTT AGC |  |  |
|  |  |  |  |  |  |
| α-tubulin | *tub****^†^** | AY326430 | F: GAC ATC ACC AAT GCC TGC TTC | 96.7 | 97.1 |
|  |  |  | R: GTG GCG ATG GCG GAG TTC |  |  |
|  |  |  |  |  |  |
| 18S rRNA | *18s****^†^** | LC203227 | F: GCA TTT ATC AGA CCC AAA ACC | 93.3 | 93.1 |
|  |  |  | R: AGT TGA TAG GGC AGA CAT TCG |  |  |
|  |  |  |  |  |  |
| Adipose triglyceride lipase | *atgl** | JX975711 | F: GTG CTT CAG TCC TGG ATG TCT TC | 94.2 |  |
|  |  |  | R: AGC CTT GCA GGT CCA TGT TGA |  |  |
|  |  |  |  |  |  |
| Calpain 1 | *capn1***^†^** | KF444899 | F: CAG AAC CAC AAC GCC GTG AAG TTT |  | 96.2 |
|  |  |  | R: AGG CAC TGG GCT TTA AGA CTC TCG |  |  |
|  |  |  |  |  |  |
| Calpain 2 | *capn2***^†^** | KF444900 | F: CAT CTA TAA GAA GAA CGA CTC GGA CAA CTC |  | 92.3 |
|  |  |  | R: TGT TGA GGC TGA AAC CTG CGT CTT TA |  |  |
|  |  |  |  |  |  |
| Calpain 3 | *capn3***^†^** | KM522785 | F: TAC GAA GAG GAT GAC GAC CCA GAG |  | 91.8 |
|  |  |  | R: GCA TCA GAG CCA CAA CGA GAG T |  |  |
|  |  |  |  |  |  |
| Calpastatin | *cast***^†^** | KM522786 | F: CCC AAA CCC GAG CCC ACC AT |  | 96.7 |
|  |  |  | R: GAC AAG AAG TCC AGA GCG TCT CCA GTA |  |  |
|  |  |  |  |  |  |
| Catalase | *cat***^†^** | JQ308823 | F: TGG TCG AGA ACT TGA AGG CTG TC |  | 98.0 |
|  |  |  | R: AGG ACG CAG AAA TGG CAG AGG |  |  |
|  |  |  |  |  |  |
| Cadherin 15 | *cdh15***^†^** | KM522781 | F: AAC GCT TAT CTG AGC TAC TCT ATC ATT GG |  | 92.2 |
|  |  |  | R: CTG GTT GTT GAT ACC GAA CAT TGT CTT G |  |  |
|  |  |  |  |  |  |
| Cytochrome c oxidase subunit 1 | *cox1****^†^** | KC217652 | F: GTC CTA CTT CTT CTG TCC CTT CCT GTT CT | 91.8 | 91.6 |
|  |  |  | R: AGG TTT CGG TCT GTA AGG AGC ATT GTA ATC |  |  |
|  |  |  |  |  |  |
| Cytochrome c oxidase subunit 2 | *cox2****^†^** | KC217653 | F: ACT GCC TAC ACA GGA CCT TGC C | 95.5 | 95.8 |
|  |  |  | R: GTC TGC TTC CAG GAG ACG GAA TTG T |  |  |
|  |  |  |  |  |  |
| Carnitine palmitoyltransferase 1a | *cpt1a****^†^** | JQ308822 | F: GTG CCT TCG TTC GTT CCA TGA TC | 92.9 | 92.5 |
|  |  |  | R: TGA TGC TTA TCT GCT GCC TGT TTG |  |  |
|  |  |  |  |  |  |
| Citrate synthase | *cs****^†^** | JX975229 | F: TCC AGG AGG TGA CGA GCC | 95.1 | 95.6 |
|  |  |  | R: GTG ACC AGC AGC CAG AAG AG |  |  |
|  |  |  |  |  |  |
| Cathepsin B | *ctsb***^†^** | KJ524457 | F: TGA TTC CCA TGT CGG TTG TC |  | 92.5 |
|  |  |  | R: GGG TCT ACT GCC ATT CAC AT |  |  |
|  |  |  |  |  |  |
| Cathepsin D | *ctsd***^†^** | AF036319 | F: CAC ACT GGG AGA CCT GCA CTA TGT CAA TG |  | 93.0 |
|  |  |  | R: ATT GCC AAC TTG AAG TCC GTC CAT ACC |  |  |
|  |  |  |  |  |  |
| Cathepsin L | *ctsl***^†^** | KM522787 | F: GGG AAC GGA TGA CCA GCC TTG T |  | 95.0 |
|  |  |  | R: CGG TGT CAT TGG CAG AGT TGT AGT TG |  |  |
|  |  |  |  |  |  |
| Cathepsin S | *ctss***^†^** | KM522788 | F: CCA CAT GGG AGA CCT GAC ACC AGA GGA GAT |  | 96.0 |
|  |  |  | R: TCA GTG GGA GGA ATG AAT GTG GCG AAA GAC |  |  |
|  |  |  |  |  |  |
| Superoxide dismutase [Cu-Zn] | *cu-zn-sod / sod1** | JQ308832 | F: TCA CGG ACA AGA TGC TCA CTC TC | 92.3 |  |
|  |  |  | R: GGT TCT GCC AAT GAT GGA CAA GG |  |  |
|  |  |  |  |  |  |
| Cholesterol 7-alpha-monooxygenase | *cyp7a1** | KX122017 | F: CCC TGC TAT TAA AGT CCC ACC TCT | 97.1 |  |
|  |  |  | R: ATC GTA GGT AGG CTG GAG GAT TC |  |  |
|  |  |  |  |  |  |
| Elongation of very long chain fatty acids 1 | *elovl1** | JX975700 | F: CTT CCT ACA CAT CTT CCA CCA CTC | 98.3 |  |
|  |  |  | R: CCA TTC CAC CAG GAG CAA AGG |  |  |
|  |  |  |  |  |  |
| Elongation of very long chain fatty acids 4 | *elovl4** | JX975701 | F: CGG TGG CAA TCA TCT TCC | 94.8 |  |
|  |  |  | R: TCA ACT GGC TGT CTG TGT |  |  |
|  |  |  |  |  |  |
| Elongation of very long chain fatty acids 5 | *elovl5** | AY660879 | F: CCT CCT GGT GCT CT ACA AT | 96.4 |  |
|  |  |  | R: GTG AGT GTC CTG GCA GTA |  |  |
|  |  |  |  |  |  |
| Elongation of very long chain fatty acids 6 | *elovl6** | JX975702 | F: GTG CTG CTC TAC TCC TGG TA | 95.8 |  |
|  |  |  | R: ACG GCA TGG ACC AAG TAG T |  |  |
|  |  |  |  |  |  |
| Fatty acid desaturase 2 | *fads2** | AY055749 | F: GCA GGC GGA GAG CGA CGG TCT GTT CC | 97.8 |  |
|  |  |  | R: AGC AGG ATG TGA CCC AGG TGG AGG CAG AAG |  |  |
|  |  |  |  |  |  |
| Follistatin | *fst***^†^** | AY544167 | F: GGA CCA GAC AAA CAA CGC ATA TTG |  | 91.6 |
|  |  |  | R: CAT AGA TGA TCC CGT CGT TTC CAC |  |  |
|  |  |  |  |  |  |
| Growth hormone receptor-type 1 | *ghr1****^†^** | AF438176 | F: ACC TGT CAG CCA CCA CAT GA | 97.6 | 97.7 |
|  |  |  | R: TCG TGC AGA TCT GGG TCG TA |  |  |
|  |  |  |  |  |  |
| Growth hormone receptor-type 2 | *ghr2****^†^** | AY573601 | F: GAG TGA ACC CGG CCT GAC AG | 98.2 | 98.5 |
|  |  |  | R: GCG GTG GTA TCT GAT TCA TGG T |  |  |
|  |  |  |  |  |  |
| Glutathione peroxidase 1 | *gpx1** | DQ524992 | F: GAA GGT GGA TGT GAA TGG AAA AGA TG | 96.3 |  |
|  |  |  | R: CTG ACG GGA CTC CAA ATG ATG G |  |  |
|  |  |  |  |  |  |
| Glutathione peroxidase 4 | *gpx4****^†^** | AM977818 | F: TGC GTC TGA TAG GGT CCA CTG TC | 91.0 | 91.4 |
|  |  |  | R: GTC TGC CAG TCC TCT GTC GG |  |  |
|  |  |  |  |  |  |
| Glutathione reductase | *gr***^†^** | AJ937873 | F: TGT TCA GCC ACC CAC CCA TCG G |  | 96.6 |
|  |  |  | R: GCG TGA TAC ATC GGA GTG AAT GAA GTC TTG |  |  |
|  |  |  |  |  |  |
| Glucose-regulated protein 170 kDa | *grp170****^†^** | JQ308821 | F: CAG AGG AGG CAG ACA GCA AGA C | 95.2 | 94.9 |
|  |  |  | R: TTC TCA GAC TCA GCA TTT CCA GAT TTC |  |  |
|  |  |  |  |  |  |
| Glucose-regulated protein 94 kDa | *grp94****^†^** | JQ308820 | F: AAG GCA CAG GCT TAC CAG ACA G | 91.6 | 91.9 |
|  |  |  | R: CTT CAG CAT CAT CGC CGA CTT TC |  |  |
|  |  |  |  |  |  |
| Glucose-regulated protein 75 kDa | *grp75****^†^** | DQ524993 | F: TCC GGT GTG GAT CTG ACC AAA GAC | 96.3 | 96.2 |
|  |  |  | R: TGT TTA GGC CCA GAA GCA TCC ATG |  |  |
|  |  |  |  |  |  |
| Fatty acid binding protein, heart | *hfabp** | JQ308834 | F: CTG GGT GTG GGC TTC GCT AC | 99.1 |  |
|  |  |  | R: CTC TGT GTT CTT GAT GGT GCT CTG |  |  |
|  |  |  |  |  |  |
| Hypoxia inducible factor 1*α* | *hif1α****^†^** | JQ308830 | F: CAG ATG AGC CTC TAA CTT GTG GAC | 96.3 | 96.5 |
|  |  |  | R: TTA GCA AGA ATG GTG GCA AGA TGA G |  |  |
|  |  |  |  |  |  |
| Hepatic lipase | *hl** | EU254479 | F: TTG TAG AAG GTG AGG AAA ACT G | 94.3 |  |
|  |  |  | R: GCT CTC CAT CAG ACC ATC C |  |  |
|  |  |  |  |  |  |
| Insulin-like growth factor 1 | *igf1****^†^** | AY996779 | F: TGT CTA GCG CTC TTT CCT TTC A | 95.4 | 95.1 |
|  |  |  | R: AGA GGG TGT GGC TAC AGG AGA TAC |  |  |
|  |  |  |  |  |  |
| Insulin-like growth factor 2 | *igf2****^†^** | AY996778 | F: TGG GAT CGT AGA GGA GTG TTG T | 93.4 | 93.5 |
|  |  |  | R: CTG TAG AGA GGT GGC CGA CA |  |  |
|  |  |  |  |  |  |
| Insulin-like growth factor binding protein 1a | *igfbp1a** | KM522771 | F: ACA AAC CAA AAC AGT GCG AGT CCT C | 98.1 |  |
|  |  |  | R: CCG TTC CAA GAG TTC ACA CAC CAG |  |  |
|  |  |  |  |  |  |
| Insulin-like growth factor binding protein 1b | *igfbp1b** | MH577189 | F: GCC AAA CAG TGT GAG TCA TC | 96.1 |  |
|  |  |  | R: ATC TTC TTC CCG TTC CAG G |  |  |
|  |  |  |  |  |  |
| Insulin-like growth factor binding protein 2a | *igfbp2a** | MH577190 | F: CCA GCA AAG AGA CCA CCT | 97.6 |  |
|  |  |  | R: TCT TCA TCT CCT GCC TGT G |  |  |
|  |  |  |  |  |  |
| Insulin-like growth factor binding protein 2b | *igfbp2b** | AF377998 | F: AGC GAT GTG TCC TGA GAT AGT GAG | 95.9 |  |
|  |  |  | R: GCA CCG TGG CGT GTA GAC C |  |  |
|  |  |  |  |  |  |
| Insulin-like growth factor binding protein 3a | *igfbp3a***^†^** | MH577191 | F: ACA GGC GTG TGG AGT GTA |  | 91.9 |
|  |  |  | R: TGG TGC TGG CAG GTC AAG |  |  |
|  |  |  |  |  |  |
| Insulin-like growth factor binding protein 4 | *igfbp4** | KM658998 | F: GGC ATC AAA CAC CCG CAC AC | 99.7 |  |
|  |  |  | R: ATC CAC GCA CCA GCA CTT CC |  |  |
|  |  |  |  |  |  |
| Insulin-like growth factor binding protein 5b | *igfbp5b***^†^** | MH577194 | F: GCA AGC AGT GTA AGC CAT CTC |  | 96.8 |
|  |  |  | R: TGA ACG CCG TAC TTG TCC A |  |  |
|  |  |  |  |  |  |
| Lipoprotein lipase | *lpl** | AY495672 | F: CGT TGC CAA GTT TGT GAC CTG | 98.3 |  |
|  |  |  | R: AGG GTG TTC TGG TTG TCT GC |  |  |
|  |  |  |  |  |  |
| Superoxide dismutase [Mn] | *mn-sod / sod2****^†^** | JQ308833 | F: CCT GAC CTG ACC TAC GAC TAT GG | 94.0 | 93.6 |
|  |  |  | R: AGT GCC TCC TGA TAT TTC TCC TCT G |  |  |
|  |  |  |  |  |  |
| Myostatin/Growth differentiation factor 8 | *mstn***^†^** | AF258448 | F: AAG AGC AGA TCA TCT ACG GCA AGA TCC |  | 96.4 |
|  |  |  | R: TCA AGA GCA TCC ACA ACG GTC TAC CA |  |  |
|  |  |  |  |  |  |
| Myogenic factor 5 | *myf5***^†^** | JN034420 | F: GCA TGG TTG ACA GCA ACA GTC CAG TGT |  | 91.2 |
|  |  |  | R: TGT CTT ATC GCC CAA AGT GTC GTT CTT CAT |  |  |
|  |  |  |  |  |  |
| Myogenic factor 6 | *myf6/mrf4***^†^** | JN034421 | F: GCA GCA ATG ACA AAC CAG AGA GAC GGA ACA |  | 98.7 |
|  |  |  | R: GAG GCT GGA GGA CGC CGA AGA TTC A |  |  |
|  |  |  |  |  |  |
| Myoblast determination protein 1 | *myod1***^†^** | AF478568 | F: ATG GAG CTG TCG GAT ATC TCT TTC |  | 96.7 |
|  |  |  | R: GAA GCA GGG GTC ATC GTA GAA ATC |  |  |
|  |  |  |  |  |  |
| Myogenic determination protein 2 | *myod2***^†^** | AF478569 | F: CCA ACT GCT CTG ATG GCA TGA TGG ATT TC |  | 99.9 |
|  |  |  | R: GAC CGT TTG CTT CTC CTG GAC TCG TAT G |  |  |
|  |  |  |  |  |  |
| NADH-ubiquinone oxidoreductase chain 2 | *nd2****^†^** | KC217558 | F: TAG GTT GAA TGA CCA TCG TA | 97.3 | 97.4 |
|  |  |  | R: GGC TAA GGA GTT GAG GTT |  |  |
|  |  |  |  |  |  |
| NADH-ubiquinone oxidoreductase chain 5 | *nd5****^†^** | KC217559 | F: CCT AAA CGC CTG AGC CCT GG | 97.8 | 97.7 |
|  |  |  | R: GCT GTA AAC GAG GTG GCT AGA AGG |  |  |
|  |  |  |  |  |  |
| Proliferator-activated receptor γ coactivator 1*α* | *pgc1α** | JX975264 | F: CGT GGG ACA GGT GTA ACC AGG ACT C | 92.0 |  |
|  |  |  | R: ACC AAC CAA GGC AGC ACA CTC TAA TTC T |  |  |
|  |  |  |  |  |  |
| 85kDa calcium-independent phospholipase A2 | *pla2g6** | JX975708 | F: CGC CAA GGA ACT CGG AAA GAT GCT | 95.9 |  |
|  |  |  | R: ACC GCA CAG CCA TCA GAG TCT |  |  |
|  |  |  |  |  |  |
| Peroxisome proliferator-activated receptor α | *pparα** | AY590299 | F: TCT CTT CAG CCC ACC ATC CC | 96.8 |  |
|  |  |  | R: ATC CCA GCG TGT CGT CTC C |  |  |
|  |  |  |  |  |  |
| Peroxisomeproliferator-activated receptor γ | *pparγ** | AY590304 | F: CGC CGT GGA CCT GTC AGA GC | 93.9 |  |
|  |  |  | R: GGA ATG GAT GGA GGA GGA GGA GAT GG |  |  |
|  |  |  |  |  |  |
| Peroxiredoxin 3 | *prdx3** | GQ252681 | F: ATC AAC ACC CCA CGC AAG ACT G | 91.2 |  |
|  |  |  | R: ACC GTT TGG ATC AAT GAG GAA CAG ACC |  |  |
|  |  |  |  |  |  |
| Peroxiredoxin 5 | *prdx5** | GQ252683 | F: GAG CAC GGA ACA GAT GGC AAG G | 91.7 |  |
|  |  |  | R: TCC ACA TTG ATC TTC TTC ACG ACT CC |  |  |
|  |  |  |  |  |  |
| Stearoyl-CoA desaturase 1a | *scd1a** | JQ277703 | F: CGG AGG CGG AGG CGT TGG AGA AGA AG | 92.9 |  |
|  |  |  | R: AGG GAG ACG GCG TAC AGG GCA CCT ATA TG |  |  |
|  |  |  |  |  |  |
| Stearoyl-CoA desaturase 1b | *scd1b** | JQ277704 | F: GCT CAA TCT CAC CAC CGC CTT CAT AG | 99.1 |  |
|  |  |  | R: GCT GCC GTC GCC CGT TCT CTG |  |  |
|  |  |  |  |  |  |
| Sirtuin1 | *sirt1****^†^** | KF018666 | F: GGT TCC TAC AGT TTC ATC CAG CAG CAC ATC | 97.3 | 97.1 |
|  |  |  | R: CCT CAG AAT GGT CCT CGG ATC GGT CTC |  |  |
|  |  |  |  |  |  |
| Sirtuin2 | *sirt2****^†^** | KF018667 | F: GAA CAA TCC GAC GAC AGC AGT GAA G | 91.9 | 91.6 |
|  |  |  | R: AGG TTA CGC AGG AAG TCC ATC TCT |  |  |
|  |  |  |  |  |  |
| Uncoupling protein 1 | *ucp1** | FJ710211 | F: GCA CAC TAC CCA ACA TCA CAA G | 99.5 |  |
|  |  |  | R: CGC CGA ACG CAG AAA CAA AG |  |  |
|  |  |  |  |  |  |
| Uncoupling protein 3 | *ucp3***^†^** | EU555336 | F: AGG TGC GAC TGG CTG ACG |  | 99.3 |
|  |  |  | R: TTC GGC ATA CAA CCT CTC CAA AG |  |  |
