# Supplementary material for: Physiologically mediated responses in gilthead sea bream (Sparus aurata) fed sustainable diets: seasonal growth under warming conditions
Source: Front Physiol. 2026 Jun 30;17:1860904. doi: 10.3389/fphys.2026.1860904 (PMC13392755; doi:10.3389/fphys.2026.1860904)
Supplement: Supplementary file 3 [file Table3.docx]

Supplementary Table 3. Primers for qPCR amplification of anterior intestine transcripts.

| **Gene** | **Symbol** | **GenBank** | **Primer** | **Amplification efficiency (%)** |
| --- | --- | --- | --- | --- |
| Beta-actin | *actb* | KY388508 | F: TCC TGC GGA ATC CAT GAG A | 95.2 |
|  |  |  | R: GAC GTC GCA CTT CAT GAT GCT |  |
|  |  |  |  |  |
| Elongation factor 1α | *ef1a* | AF184170 | F: CCC GCC TCT GTT GCC TTC G | 96.4 |
|  |  |  | R: CAG CAG TGT GGT TCC GTT AGC |  |
|  |  |  |  |  |
| α-tubulin | *tub* | AY326430 | F: GAC ATC ACC AAT GCC TGC TTC | 96.8 |
|  |  |  | R: GTG GCG ATG GCG GAG TTC |  |
|  |  |  |  |  |
| 18S rRNA | *18s* | LC203227 | F: GCA TTT ATC AGA CCC AAA ACC | 93.0 |
|  |  |  | R: AGT TGA TAG GGC AGA CAT TCG |  |
|  |  |  |  |  |
| Intestinal-type alkaline phosphatase | *alpi* | KF857309 | F: CCG CTA TGA GTT GGA CCG TGA T | 98.7 |
|  |  |  | R: GCT TTC TCC ACC ATC TCA GTA AGG G |  |
|  |  |  |  |  |
| C-C chemokine receptor type 3 | *ccr3* | KF857317 | F: CTA CAT CAG CAT CAC CAT ACG CAT CCT | 95.4 |
|  |  |  | R: TGG CAC GGC ACT TCT CCT TCA |  |
|  |  |  |  |  |
| C-C chemokine receptor type 9 | *ccr9* | KF857318 | F: TCC CTG AGT TAA TCT TCG CCC AAG TG | 95.3 |
|  |  |  | R: TGT TGT ATT CGT TGT TCC AGT AGA CCA GAG |  |
|  |  |  |  |  |
| C-C chemokine receptor type 11 | *ccr11* | KF857319 | F: GCT ACG ATT ACA GTT ATG AA | 96.8 |
|  |  |  | R: TAG ATG ATT GGG AGG AAG |  |
|  |  |  |  |  |
| CD209 antigen-like protein D | *cd209d* | KF857327 | F: CGC CAC GAG CAT GAG GAC AA | 92.0 |
|  |  |  | R: TCT TGC CAG AAT CCA TCA CCA TCC A |  |
|  |  |  |  |  |
| CD302 antigen | *cd302* | KF857328 | F: GGA CCA GAG GAA GAG CAC ATC | 98.1 |
|  |  |  | R: GAC CAG GGC GGA CAT CAG |  |
|  |  |  |  |  |
| Cluster of differentiation 4 | *cd4-1* | AM489485 | F: TCC TCC TCC TCG TCC TCG TT | 96.5 |
|  |  |  | R: GGT GTC TCA TCT TCC GCT GTC T |  |
|  |  |  |  |  |
| Cluster of differentiation 8*β* | *cd8β* | KX231275 | F: CCG AAA TGT GGA AGA CTG GAA CTC | 98.4 |
|  |  |  | R: CTT TGG AGG TAA GGT TGG AGG GAT |  |
|  |  |  |  |  |
| Cadherin-1 | *cdh1* | KF861995 | F: TGC TCC ATA CAG CGT CAC CTT ACA | 91.3 |
|  |  |  | R: CTC GTT CAT CCT AGC CGT CCA GTT |  |
|  |  |  |  |  |
| Cadherin-17 | *cdh17* | KF861996 | F: GAT GCC CGC AAC CCA GAG | 96.1 |
|  |  |  | R: CCG TTG ATT CAC TGC CGT AGA C |  |
|  |  |  |  |  |
| C-C chemokine CK8 / C-C motif chemokine 20 | *ck8 / ccl20* | GU181393 | F: CCG TCC TCA TCT GCT TCA TAC T | 94.8 |
|  |  |  | R: GCT CTG CCG TTG ATG GAA C |  |
|  |  |  |  |  |
| Claudin-12 | *cldn12* | KF861992 | F: CTC TCA GGG CTA CAC ATC TAC CTA TGC | 97.0 |
|  |  |  | R: ACA TTC GTG AGC GGC TGG AG |  |
|  |  |  |  |  |
| Claudin-15 | *cldn15* | KF861993 | F: CCG ATT GTG GAA GTA GTG GCT CTG GT | 97.0 |
|  |  |  | R: CAG CAT CAC CCA ACC GAC GAA CC |  |
|  |  |  |  |  |
| Macrophage colony-stimulating factor 1 receptor 1 | *csf1r1* | AM050293 | F: TTG CGT GTG GTG AGG AAG GAA GGT | 98.1 |
|  |  |  | R: AGC AGG CAG GGC AGC AGG TA |  |
|  |  |  |  |  |
| Gap junction Cx32.2 protein | *cx32.2* | KF862000 | F: CGA GGT GTT CTA TCT GCT CTG TA | 98.6 |
|  |  |  | R: CTT GTG GGT GCG AGT CCT |  |
|  |  |  |  |  |
| Coxsackievirus and adenovirus receptor homolog | *cxadr* | KF861998 | F: CAT CAG AGG ACT ACG AGA GG | 95.0 |
|  |  |  | R: CAT CTT GGC AGC ATT TGG T |  |
|  |  |  |  |  |
| Desmoplakin | *dsp* | KF861999 | F: GCA GAA GGA GCA CGA GAC CATC | 95.9 |
|  |  |  | R: GGG TGT TCT TGT CGC AGG TGA A |  |
|  |  |  |  |  |
| Liver type fatty acid-binding protein | *fabp1* | KF857311 | F: GTC CTC GTC AAC ACC TTC ACC AT | 94.1 |
|  |  |  | R: CGC CTT CAT CTT CTC GCC AGT |  |
|  |  |  |  |  |
| Intestinal fatty acid-binding protein | *fabp2* | KF857310 | F: CGA GCA CAT TCC GCA CCA AAG | 91.4 |
|  |  |  | R: CCC ACG CAC CCG AGA CTT C |  |
|  |  |  |  |  |
| Ileal fatty acid-binding protein | *fabp6* | KF857312 | F: ACC CAG GAC GGC AAT ACC | 98.9 |
|  |  |  | R: CGA CGG TGA AGT TGT TGG T |  |
|  |  |  |  |  |
| Fucolectin | *fcl* | KF857331 | F: CCA TAC TGC TGA ACA GAC CAA CC | 94.3 |
|  |  |  | R: TGA TGG AGG TGA CGA TGT AGG A |  |
|  |  |  |  |  |
| Transcription factor HES-1-B | *hes1-b* | KF857344 | F: GCC TGC CGA TAT GAT GGA A | 92.6 |
|  |  |  | R: GGA GTT GTG TTC ATG CTT GC |  |
|  |  |  |  |  |
| Immunoglobulin M | *igm* | JQ811851 | F: ACC TCA GCG TCC TTC AGT GTT TAT GAT GCC | 95.3 |
|  |  |  | R: CAG CGT CGT CGT CAA CAA GCC AAG C |  |
|  |  |  |  |  |
| Immunoglobulin T membrane-bound form | *igtm* | KX599201 | F: AGA CGA TGC CAG TGA AGA GGA TGA GT | 98.7 |
|  |  |  | R: CGA AGG AGG AGG CTG TGG ACC A |  |
|  |  |  |  |  |
| Interleukin-1 beta | *il1β* | AJ419178 | F: GCG ACC TAC CTG CCA CCT ACA CC | 94.9 |
|  |  |  | R: TCG TCC ACC GCC TCC AGA TGC |  |
|  |  |  |  |  |
| Interleukin-6 | *il6* | EU244588 | F: TCT TGA AGG TGG TGC TGG AAG TG | 91.6 |
|  |  |  | R: AAG GAC AAT CTG CTG GAA GTG AGG |  |
|  |  |  |  |  |
| Interleukin-7 | *il7* | JX976618 | F: CTA TCT CTG TCC CTG TCC TGT GA | 98.6 |
|  |  |  | R: TGC GGA TGG TTG CCT TGT AAT |  |
|  |  |  |  |  |
| Interleukin-8 | *il8* | JX976619 | F: CAG CAG AGT CTT CAT CGT CAC TAT TG | 99.2 |
|  |  |  | R: AGG CTC GCT TCA CTG ATG G |  |
|  |  |  |  |  |
| Interleukin-10 | *il10* | JX976621 | F: AAC ATC CTG GGC TTC TAT CTG | 99.8 |
|  |  |  | R: GTG TCC TCC GTC TCA TCT G |  |
|  |  |  |  |  |
| Interleukin-12 subunit beta | *il12β* | JX976624 | F: ATT CCC TGT GTG GTG GCT GCT | 98.5 |
|  |  |  | R: GCT GGC ATC CTG GCA CTG AAT |  |
|  |  |  |  |  |
| Interleukin-15 | *il15* | JX976625 | F: GAG ACC AGC GAG CGA AAG GCA TCC | 98.9 |
|  |  |  | R: GCC AGA ACA GGT TAC AGG TTG ACA GGA A |  |
|  |  |  |  |  |
| Interleukin-34 | *il34* | JX976629 | F: TCT GTC TGC CTG CTG GTA G | 95.4 |
|  |  |  | R: ATG CTG GCT GGT GTC TGG |  |
|  |  |  |  |  |
| Krueppel-like factor 4 | *klf4* | KF857346 | F: ACA TCA CCG CAC GCA CAC | 97.8 |
|  |  |  | R: AAC CAC AGC CCT CCC AGT C |  |
|  |  |  |  |  |
| Galectin-1 | *lgals1* | KF862003 | F: GTG TGA GGA GGT CCG TGA TG | 96.0 |
|  |  |  | R: ACT GTA GAG CCG TCC GAT AGG |  |
|  |  |  |  |  |
| Galectin-8 | *lgals8* | KF862004 | F: GGC GGT GAA CGG CGG TCA | 93.3 |
|  |  |  | R: GCT CCA GCT CCA GTC TGT GTT GAT AC |  |
|  |  |  |  |  |
| Macrophage mannose receptor 1 | *mrc1* | KF857326 | F: CTT CCG ACC GTA CCT GTA CCT ACT CA | 91.9 |
|  |  |  | R: CGA TTC CAG CCT TCC GCA CAC TTA |  |
|  |  |  |  |  |
| Mucin 2 | *muc2* | JQ277710 | F: ACG CTT CAG CAA TCG CAC CAT | 94.8 |
|  |  |  | R: CCA CAA CCA CAC TCC TCC ACA T |  |
|  |  |  |  |  |
| Mucin 13 | *muc13* | JQ277713 | F: TTC AAA CCC GTG TGG TCC AG | 95.8 |
|  |  |  | R: GCA CAA GCA GAC ATA GTT CGG ATA T |  |
|  |  |  |  |  |
| Proliferating cell nuclear antigen | *pcna* | KF857335 | F: CGT ATC TGC CGT GAC CTG T | 99.3 |
|  |  |  | R: AGA ACT TGA CTC CGT CCT TGG |  |
|  |  |  |  |  |
| Tight junction protein ZO-1 | *tjp1* | KF861994 | F: AAG CAG TAT TAC GGT GAC TCA | 91.3 |
|  |  |  | R: TGC ATC CCT GGC TTG TAG |  |
|  |  |  |  |  |
| Toll-like receptor 2 | *tlr2* | KF857323 | F: CAT CTG CGA CTC TCC TCT CTT CCT | 92.0 |
|  |  |  | R: ATT CAA CAA TGG AGC GGT GGA CTT |  |
|  |  |  |  |  |
| Toll-like receptor 5 | *tlr5* | KF857324 | F: TCG CCA ATC TGA CGG ACC TGA G | 94.5 |
|  |  |  | R: CAG AAC GCC GAT GTG GTT GTA AGA C |  |
|  |  |  |  |  |
| Toll-like receptor 9 | *tlr9* | AY751797 | F: GCC TTC CTT GTC TGC TCT TTC T | 98.0 |
|  |  |  | R: GCC GTA GAG GTG CTT CAG TAG |  |
|  |  |  |  |  |
| Tumor necrosis factor alpha | *tnfα* | AJ413189 | F: CAG GCG TCG TTC AGA GTC TC | 98.3 |
|  |  |  | R: CTG TGG CTG AGA GGT GTG AG |  |
|  |  |  |  |  |
