# Supplementary material for: Physiologically mediated responses in gilthead sea bream (Sparus aurata) fed sustainable diets: seasonal growth under warming conditions
Source: Front Physiol. 2026 Jun 30;17:1860904. doi: 10.3389/fphys.2026.1860904 (PMC13392755; doi:10.3389/fphys.2026.1860904)
Supplement: Supplementary file 4 [file Table4.docx]

Supplementary Table 4. Results of the two-way ANOVA evaluating the effects of diet (CTRL, PAP, ALT) and sampling time (T_2_, November 2022; T3, February 2023) on liver weight, viscera weight, HSI, VSI and muscle fat content. Data are expressed as mean ± SEM (n = 10–16 per experimental condition). Statistically significant effects of time, diet, or their interaction are highlighted in bold.

|  |  | T2 | | |  |  | T3 | | |  |  | Two-Way ANOVA | | |
| --- | --- | --- | --- | --- | --- | --- | --- | --- | --- | --- | --- | --- | --- | --- |
|  |  | CTRL | PAP | ALT | *p* |  | CTRL | PAP | ALT | *p* |  | Time | Diet | Time x Diet |
| Liver weight (g) |  | 5.15±0.27 | 5.39±0.34 | 5.92±0.38 | 0.257 |  | 7.99±0.53 | 7.72±0.50 | 8.28±0.39 | 0.613 |  | **<0.001** | 0.353 | 0.839 |
| Viscera weight (g) |  | 19.67±0.92 | 18.44±0.83 | 19.78±0.85 | 0.491 |  | 22.96±0.95 | 22.85±1.18 | 22.66±0.74 | 0.975 |  | **<0.001** | 0.787 | 0.679 |
| HSI (%) 6 |  | 1.70±0.08 | 1.90±0.09 | 1.99±0.11 | 0.095 |  | 2.17±0.12 | 2.26±0.10 | 2.34±0.09 | 0.482 |  | **<0.001** | 0.088 | 0.733 |
| VSI (%) 5 |  | 6.52±0.27 | 6.53±0.19 | 6.47±0.18 | 0.980 |  | 6.44±0.18 | 6.84±0.17 | 6.64±0.18 | 0.305 |  | 0.401 | 0.745 | 0.780 |
| Muscle fat (%) |  | 10.08±0.46 | 11.30±0.54 | 11.37±0.65 | 0.196 |  | 8.49±0.58 | 10.00±0.54 | 9.25±0.59 | 0.187 |  | **<0.001** | 0.067 | 0.764 |
