# Supplementary material for: Physiologically mediated responses in gilthead sea bream (Sparus aurata) fed sustainable diets: seasonal growth under warming conditions
Source: Front Physiol. 2026 Jun 30;17:1860904. doi: 10.3389/fphys.2026.1860904 (PMC13392755; doi:10.3389/fphys.2026.1860904)
Supplement: Supplementary file 5 [file Table5.docx]

|  | T1 (July 2022) | | |  |  | T2 (November 2022) | | |  |  | T3 (February 2023) | | |  |
| --- | --- | --- | --- | --- | --- | --- | --- | --- | --- | --- | --- | --- | --- | --- |
|  | CTRL | PAP | ALT | *p* |  | CTRL | PAP | ALT | *p* |  | CTRL | PAP | ALT | *p* |
| *ghr1* | 1.01±0.07 | 1.01±0.15 | 1.04±0.11 | 0.975 |  | 1.08±0.08 | 1.39±0.17 | 1.23±0.15 | 0.359 |  | 1.08±0.12 | 1.32±0.09 | 1.25±0.13 | 0.389 |
| *ghr2* | 2.15±0.24^a^ | 1.61±0.18^b^ | 1.50±0.13^b^ | **0.043** |  | 1.36±0.25^a^ | 0.78±0.14^b^ | 0.80±0.10^b^ | **0.038** |  | 1.02±0.08 | 1.06±0.09 | 1.06±0.09 | 0.919 |
| *igf1* | 7.20±0.53^a^ | 4.79±0.52^b^ | 5.51±0.43^b^ | **0.009** |  | 4.90±0.44^a^ | 3.31±0.20^b^ | 3.63±0.24^b^ | **0.004** |  | 5.34±0.38 | 5.69±0.43 | 5.56±0.43 | 0.834 |
| *igf2* | 2.75±0.36^a^ | 1.83±0.18 ^b^ | 2.14±0.16^ab^ | **0.046** |  | 3.73±0.49^a^ | 1.62±0.32^b^ | 2.23±0.22^b^ | **0.001** |  | 3.21±0.28 | 3.87±0.28 | 3.63±0.33 | 0.323 |
| *igfbp1a* | 0.07±0.02 | 0.07±0.02 | 0.07±0.02 | 0.920 |  | 0.03±0.01 | 0.03±0.01 | 0.02±0.00 | 0.208 |  | 0.04±0.01 | 0.03±0.00 | 0.04±0.00 | 0.480 |
| *igfbp1b* | 1.71±0.29 | 1.68±0.35 | 2.07±0.80 | 0.860 |  | 0.35±0.07 | 0.36±0.06 | 0.44±0.23 | 0.366 |  | 1.19±0.40 | 0.52±0.11 | 0.73±0.15 | 0.403 |
| *igfbp2a* | 1.09±0.13 | 0.79±0.06 | 0.84±0.06 | 0.065 |  | 0.77±0.06^a^ | 0.62±0.05^ab^ | 0.49±0.09^b^ | **0.047** |  | 0.75±0.09^b^ | 1.03±0.08^a^ | 1.11±0.06^a^ | **0.005** |
| *igfbp2b* | 1.90±0.16 | 1.71±0.20 | 1.75±0.13 | 0.718 |  | 1.36±0.11^a^ | 1.00±0.09^b^ | 0.75±0.12^b^ | **0.004** |  | 1.15±0.09 | 1.06±0.09 | 1.13±0.07 | 0.761 |
| *igfbp4* | 0.58±0.04 | 0.58±0.02 | 0.64±0.04 | 0.318 |  | 0.63±0.05 | 0.64±0.05 | 0.61±0.05 | 0.936 |  | 0.76±0.09^ab^ | 0.85±0.06^a^ | 0.54±0.05^b^ | **0.011** |
| *elovl1* | 4.46±0.42 | 4.65±0.38 | 4.28±0.40 | 0.759 |  | 6.54±0.51 | 5.90±0.29 | 6.18±0.31 | 0.493 |  | 11.38±0.56 | 11.88±0.71 | 12.07±1.09 | 0.858 |
| *elovl4* | 0.16±0.02 | 0.17±0.02 | 0.19±0.02 | 0.598 |  | 0.18±0.01 | 0.16±0.01 | 0.15±0.01 | 0.297 |  | 0.21±0.02 | 0.23±0.02 | 0.20±0.01 | 0.424 |
| *elovl5* | 1.25±0.29 | 1.33±0.58 | 1.73±0.41 | 0.393 |  | 0.80±0.23 | 1.18±0.21 | 1.01±0.20 | 0.416 |  | 0.50±0.07 | 0.84±0.24 | 0.60±0.15 | 0.816 |
| *elovl6* | 0.39±0.06 | 0.38±0.07 | 0.37±0.07 | 0.685 |  | 1.10±0.30 | 1.53±0.25 | 1.57±0.35 | 0.510 |  | 1.52±0.29^b^ | 5.18±0.71^a^ | 2.32±0.51^b^ | **<0.001** |
| *fads2* | 2.77±0.58 | 3.06±1.09 | 1.93±0.44 | 0.424 |  | 2.24±0.52 | 3.08±0.74 | 2.70±0.59 | 0.666 |  | 0.77±0.10^b^ | 3.54±0.60^a^ | 1.66±0.30^b^ | **<0.001** |
| *scd1a* | 0.12±0.02 | 0.12±0.04 | 0.12±0.03 | 0.799 |  | 0.17±0.02 | 0.27±0.06 | 0.51±0.15 | 0.064 |  | 0.60±0.10^b^ | 1.27±0.23^a^ | 0.69±0.10^b^ | **0.011** |
| *scd1b* | 0.17±0.03 | 0.12±0.05 | 0.20±0.11 | 0.429 |  | 0.27±0.11 | 0.55±0.15 | 1.33±0.49 | 0.090 |  | 2.50±0.42^b^ | 10.23±1.93^a^ | 3.83±0.71^b^ | **0.003** |
| *hl* | 7.49±0.54 | 6.93±0.46 | 6.50±0.47 | 0.362 |  | 3.06±0.31 | 2.91±0.23 | 2.83±0.20 | 0.814 |  | 2.03±0.22 | 1.82±0.13 | 1.74±0.22 | 0.552 |
| *lpl* | 1.43±0.18 | 1.67±0.22 | 1.06±0.10 | 0.051 |  | 1.53±0.15 | 1.38±0.18 | 1.13±0.16 | 0.265 |  | 1.05±0.11 | 1.23±0.15 | 1.33±0.31 | 0.531 |
| *atgl* | 0.15±0.04 | 0.11±0.03 | 0.09±0.01 | 0.426 |  | 0.23±0.07^a^ | 0.10±0.03^b^ | 0.09±0.02^b^ | **0.040** |  | 0.11±0.03 | 0.08±0.02 | 0.16±0.05 | 0.359 |
| *pla2g6* | 0.08±0.01 | 0.07±0.01 | 0.09±0.01 | 0.196 |  | 0.10±0.01 | 0.12±0.02 | 0.10±0.01 | 0.971 |  | 0.22±0.02 | 0.17±0.02 | 0.18±0.02 | 0.088 |
| *cyp7a1* | 1.48±0.21 | 1.32±0.24 | 1.13±0.14 | 0.460 |  | 1.61±0.34^a^ | 0.86±0.10^b^ | 1.09±0.13^ab^ | **0.045** |  | 2.65±0.59 | 2.57±0.29 | 2.23±0.43 | 0.751 |
| *pparα* | 1.35±0.13 | 1.40±0.13 | 1.08±0.09 | 0.133 |  | 1.61±0.23 | 1.53±0.10 | 1.19±0.13 | 0.073 |  | 1.31±0.17 | 1.50±0.13 | 1.25±0.10 | 0.342 |
| *pparγ* | 0.36±0.03 | 0.37±0.02 | 0.38±0.03 | 0.864 |  | 0.25±0.02 | 0.26±0.02 | 0.27±0.02 | 0.654 |  | 0.61±0.05 | 0.53±0.03 | 0.52±0.04 | 0.231 |
| *hif1α* | 0.47±0.02 | 0.44±0.02 | 0.51±0.04 | 0.252 |  | 0.37±0.02 | 0.36±0.02 | 0.37±0.02 | 0.711 |  | 0.52±0.04 | 0.49±0.02 | 0.48±0.03 | 0.601 |
| *cpt1a* | 0.38±0.03 | 0.38±0.05 | 0.37±0.04 | 0.968 |  | 0.68±0.04^a^ | 0.42±0.03^b^ | 0.35±0.04^b^ | **<0.001** |  | 0.70±0.04 | 0.61±0.04 | 0.62±0.05 | 0.285 |
| *hfabp* | 18.59±3.63 | 18.43±3.23 | 11.96±1.79 | 0.197 |  | 21.32±1.97 | 22.93±3.01 | 21.27±2.63 | 0.879 |  | 21.29±1.64^b^ | 32.85±3.31^a^ | 26.91±2.14^ab^ | **0.025** |
| *cs* | 0.68±0.05 | 0.57±0.07 | 0.50±0.03 | 0.065 |  | 0.51±0.05 | 0.48±0.02 | 0.49±0.02 | 0.749 |  | 0.91±0.04 | 0.81±0.05 | 0.95±0.07 | 0.081 |
| *nd2* | 17.51±0.97 | 16.96±1.79 | 18.92±2.13 | 0.708 |  | 19.09±3.32 | 16.41±1.10 | 19.22±1.75 | 0.544 |  | 31.02±3.19^a^ | 15.97±0.83^b^ | 20.31±2.39^b^ | **<0.001** |
| *nd5* | 8.00±0.47 | 6.81±0.86 | 7.13±0.74 | 0.484 |  | 5.04±0.95 | 4.57±0.46 | 6.03±0.66 | 0.099 |  | 7.67±0.86^a^ | 4.43±0.24^b^ | 8.13±0.92^a^ | **<.0.001** |
| *cox1* | 71.82±6.94 | 63.00±6.19 | 72.81±8.09 | 0.584 |  | 44.29±9.17 | 40.34±4.82 | 46.49±4.26 | 0.392 |  | 73.32±7.48^a^ | 40.29±3.76^b^ | 63.40±7.22^a^ | **0.003** |
| *cox2* | 19.91±1.57 | 17.63±1.49 | 19.70±1.75 | 0.566 |  | 10.68±1.41 | 10.39±0.65 | 12.30±1.18 | 0.399 |  | 16.60±1.90 | 11.51±0.96 | 14.37±1.70 | 0.085 |
| *pgc1α* | 0.05±0.01 | 0.06±0.02 | 0.04±0.01 | 0.437 |  | 0.06±0.01 | 0.04±0.01 | 0.03±0.01 | 0.168 |  | 0.07±0.01 | 0.06±0.01 | 0.08±0.01 | 0.286 |
| *sirt1* | 0.05±0.00 | 0.05±0.00 | 0.05±0.00 | 0.722 |  | 0.06±0.00 | 0.06±0.00 | 0.05±0.00 | 0.689 |  | 0.08±0.01 | 0.09±0.01 | 0.09±0.01 | 0.565 |
| *sirt2* | 0.16±0.01 | 0.14±0.01 | 0.14±0.01 | 0.068 |  | 0.14±0.01 | 0.13±0.00 | 0.14±0.01 | 0.304 |  | 0.16±0.00 | 0.17±0.00 | 0.17±0.00 | 0.270 |
| *ucp1* | 12.11±0.97 | 11.44±1.80 | 12.81±1.75 | 0.917 |  | 4.81±0.78 | 5.04±0.59 | 4.55±0.38 | 0.825 |  | 4.07±0.60^b^ | 5.69±0.41^a^ | 4.12±0.45^b^ | **0.047** |
| *gpx1* | 1.54±0.17 | 1.16±0.11 | 1.19±0.08 | 0.071 |  | 1.22±0.13 | 0.94±0.11 | 0.89±0.10 | 0.112 |  | 0.90±0.06^ab^ | 1.11±0.09^a^ | 0.77±0.07^b^ | **0.007** |
| *gpx4* | 6.07±0.82 | 4.46±0.79 | 5.13±0.46 | 0.297 |  | 4.33±0.70 | 4.00±0.47 | 4.75±0.47 | 0.553 |  | 14.80±0.80 | 13.26±0.76 | 14.76±1.16 | 0.424 |
| *prdx3* | 0.54±0.03 | 0.48±0.04 | 0.46±0.05 | 0.081 |  | 0.36±0.04 | 0.32±0.02 | 0.37±0.05 | 0.763 |  | 0.82±0.08 | 0.85±0.05 | 0.74±0.07 | 0.484 |
| *prdx5* | 0.40±0.01 | 0.33±0.03 | 0.37±0.03 | 0.080 |  | 0.64±0.04 | 0.48±0.05 | 0.51±0.04 | 0.084 |  | 1.23±0.10 | 1.13±0.09 | 1.06±0.08 | 0.441 |
| *cu-zn-sod / sod1* | 4.85±0.47 | 3.54±0.27 | 3.83±0.43 | 0.080 |  | 3.53±0.29 | 3.49±0.25 | 3.62±0.27 | 0.932 |  | 4.92±0.18 | 4.95±0.32 | 4.78±0.23 | 0.731 |
| *mn-sod / sod2* | 0.55±0.03 | 0.49±0.03 | 0.58±0.04 | 0.204 |  | 0.75±0.07 | 0.70±0.08 | 0.87±0.12 | 0.381 |  | 1.47±0.12 | 1.13±0.10 | 1.51±0.14 | 0.056 |
| *grp170* | 0.60±0.03 | 0.87±0.10 | 0.80±0.11 | 0.190 |  | 0.90±0.22 | 0.71±0.08 | 0.79±0.12 | 0.929 |  | 3.87±0.86^a^ | 1.71±0.14^b^ | 3.23±0.42^b^ | **0.011** |
| *grp94* | 2.44±0.27 | 3.68±0.47 | 3.15±0.30 | 0.072 |  | 1.70±0.43 | 1.27±0.08 | 1.64±0.30 | 0.889 |  | 4.37±2.05 | 1.42±0.22 | 2.51±0.67 | 0.725 |
| *grp75* | 0.32±0.03 | 0.37±0.03 | 0.39±0.04 | 0.109 |  | 0.43±0.05 | 0.41±0.03 | 0.48±0.06 | 0.750 |  | 0.78±0.13^ab^ | 0.61±0.05^b^ | 1.12±0.19^a^ | **0.039** |

Supplementary Table 5. Relative gene expression of liver mRNA transcripts of fish the CTRL and experimental (PAP, ALT) diets over the production cycle. Values are the mean ± SEM of 10-16 fish. Data are in reference to the expression level of *ghr1* in fish from the CTRL diet at the corresponding time with an arbitrary value of 1. Nutritionally regulated genes at a given sampling time are in bold. Different letters indicate statistically significant differences (Holm-Sidak post hoc test, *P* < 0.05) among groups at a given sampling time.
