# Supplementary material for: Physiologically mediated responses in gilthead sea bream (Sparus aurata) fed sustainable diets: seasonal growth under warming conditions
Source: Front Physiol. 2026 Jun 30;17:1860904. doi: 10.3389/fphys.2026.1860904 (PMC13392755; doi:10.3389/fphys.2026.1860904)
Supplement: Supplementary file 6 [file Table6.docx]

Supplementary Table 6. Relative gene expression of white skeletal muscle mRNA transcripts of fish fed three experimental diets (CTRL, PAP, ALT) along three periods of time (t_1_, t_2_, t_3_). Values are the mean ± SEM of 10-16 fish per experimental condition. All data from t_1_, t_2_ and t_3_ are in reference to the expression level of *ghr1* in fish from CTRL group of each time with an arbitrary value of 1. Different letters indicate statistically significant differences (Holm-Sidak post hoc test, *P* < 0.05). Differentially expressed genes are in bold.

|  | t_1_ | | |  |  | t_2_ | | |  |  | t_3_ | | |  |
| --- | --- | --- | --- | --- | --- | --- | --- | --- | --- | --- | --- | --- | --- | --- |
|  | CTRL | PAP | ALT | *p* |  | CTRL | PAP | ALT | *p* |  | CTRL | PAP | ALT | *p* |
| *ghr1* | 1.03±0.12 | 0.87±0.06 | 0.85±0.09 | 0.319 |  | 1.03±0.10 | 1.32±0.24 | 1.41±0.14 | 0.274 |  | 1.11±0.16^b^ | 0.90±0.08^b^ | 1.67±0.22^a^ | **0.013** |
| *ghr2* | 0.62±0.05 | 0.71±0.10 | 0.56±0.07 | 0.422 |  | 0.22±0.05 | 0.17±0.03 | 0.23±0.03 | 0.429 |  | 0.76±0.24^ab^ | 0.64±0.08^b^ | 1.27±0.20^a^ | **0.046** |
| *igf1* | 0.09±0.01^a^ | 0.04±0.01^b^ | 0.06±0.01^ab^ | **0.026** |  | 0.02±0.00 | 0.02±0.01 | 0.01±0.00 | 0.186 |  | 0.03±0.00^a^ | 0.04±0.00^a^ | 0.01±0.00^b^ | **<0.001** |
| *igf2* | 0.43±0.03 | 0.39±0.03 | 0.36±0.02 | 0.140 |  | 0.14±0.01 | 0.15±0.01 | 0.14±0.01 | 0.720 |  | 0.66±0.10 | 0.57±0.03 | 0.57±0.06 | 0.973 |
| *igfbp3a* | 2.30±0.35 | 1.83±0.21 | 1.56±0.15 | 0.102 |  | 0.49±0.08^b^ | 0.55±0.06^ab^ | 0.75±0.07^a^ | **0.035** |  | 0.45±0.03 | 0.59±0.04 | 0.54±0.05 | 0.055 |
| *igfbp5b* | 1.44±0.14^a^ | 1.06±0.09^b^ | 0.99±0.10^b^ | **0.023** |  | 0.96±0.11 | 0.98±0.06 | 0.96±0.07 | 0.991 |  | 2.11±0.25^b^ | 2.79±0.10^a^ | 2.21±0.11^b^ | **0.005** |
| *myod1* | 3.20±0.36 | 3.27±0.36 | 2.59±0.21 | 0.218 |  | 2.07±0.24 | 2.16±0.34 | 2.01±0.17 | 0.917 |  | 2.62±0.26 | 3.12±0.18 | 3.11±0.30 | 0.299 |
| *myod2* | 1.90±0.29 | 1.70±0.19 | 2.02±0.11 | 0.442 |  | 0.39±0.07^b^ | 0.49±0.07^ab^ | 0.68±0.05^a^ | **0.006** |  | 0.49±0.06 | 0.78±0.12 | 0.43±0.11 | 0.055 |
| *myf5* | 0.20±0.01 | 0.17±0.01 | 0.18±0.01 | 0.071 |  | 0.05±0.00 | 0.05±0.00 | 0.05±0.00 | 0.063 |  | 0.08±0.00 | 0.09±0.01 | 0.09±0.02 | 0.589 |
| *myf6/mrf4* | 0.18±0.01^a^ | 0.15±0.01^b^ | 0.15±0.01^b^ | **0.012** |  | 0.11±0.00 | 0.10±0.01 | 0.10±0.01 | 0.104 |  | 0.27±0.04 | 0.20±0.01 | 0.24±0.03 | 0.204 |
| *mstn* | 1.83±0.41 | 1.84±0.26 | 1.26±0.21 | 0.132 |  | 0.58±0.19 | 0.58±0.10 | 0.57±0.22 | 0.638 |  | 0.79±0.23 | 0.46±0.10 | 0.58±0.14 | 0.464 |
| *fst* | 0.44±0.04^a^ | 0.23±0.03^b^ | 0.28±0.05^b^ | **0.007** |  | 0.07±0.02 | 0.05±0.01 | 0.06±0.01 | 0.902 |  | 0.22±0.04 | 0.19±0.01 | 0.22±0.03 | 0.771 |
| *cdh15* | 0.22±0.01 | 0.23±0.02 | 0.26±0.02 | 0.298 |  | 0.07±0.01 | 0.08±0.01 | 0.07±0.01 | 0.634 |  | 0.20±0.01^a^ | 0.24±0.02^a^ | 0.13±0.02^b^ | **0.001** |
| *hif1α* | 0.72±0.03 | 0.69±0.03 | 0.66±0.02 | 0.399 |  | 0.21±0.02 | 0.21±0.01 | 0.19±0.01 | 0.376 |  | 0.68±0.07^a^ | 0.48±0.03^b^ | 0.53±0.03^ab^ | **0.035** |
| *cpt1a* | 1.57±0.10 | 1.58±0.09 | 1.30±0.09 | 0.071 |  | 1.51±0.10 | 1.52±0.15 | 1.18±0.16 | 0.165 |  | 3.52±0.45^a^ | 2.16±0.18^b^ | 4.18±0.58^a^ | **0.005** |
| *cs* | 8.60±0.35^a^ | 7.09±0.29^b^ | 6.54±0.34^b^ | **<0.001** |  | 5.09±0.38 | 4.79±0.45 | 4.74±0.32 | 0.781 |  | 11.70±1.20 | 10.90±1.10 | 14.49±1.50 | 0.353 |
| *nd2* | 24.03±0.90 | 20.54±1.16 | 24.25±2.60 | 0.141 |  | 17.68±2.48 | 16.98±2.09 | 13.10±1.08 | 0.170 |  | 36.53±3.36 | 30.01±2.94 | 30.95±3.78 | 0.212 |
| *nd5* | 11.19±0.61^a^ | 8.86±0.47^b^ | 10.70±0.77^ab^ | **0.048** |  | 7.29±1.02 | 6.20±0.70 | 5.65±0.39 | 0.953 |  | 10.07±0.44 | 7.99±0.60 | 9.98±1.24 | 0.112 |
| *cox1* | 159.01±10.25 | 134.77±6.07 | 143.16±8.20 | 0.151 |  | 88.73±9.04 | 84.96±8.81 | 72.60±6.37 | 0.132 |  | 114.12±6.96^a^ | 81.26±4.42^b^ | 113.04±14.90^ab^ | **0.007** |
| *cox2* | 33.62±1.99^a^ | 27.00±1.26^b^ | 28.77±1.79^ab^ | **0.042** |  | 14.50±1.73 | 13.29±1.51 | 10.80±0.86 | 0.149 |  | 21.78±1.31^a^ | 14.23±0.71^b^ | 19.55±2.31^ab^ | **0.002** |
| *ucp3* | 3.11±0.44 | 3.22±0.32 | 2.29±0.28 | 0.104 |  | 4.83±0.22^a^ | 3.11±0.22^b^ | 2.36±0.24^c^ | **<0.001** |  | 10.51±1.80^a^ | 5.72±0.36^b^ | 12.66±3.24^ab^ | **0.014** |
| *sirt1* | 0.17±0.01 | 0.14±0.01 | 0.15±0.01 | 0.071 |  | 0.09±0.01 | 0.09±0.00 | 0.10±0.01 | 0.617 |  | 0.27±0.04^ab^ | 0.19±0.01^b^ | 0.27±0.02^a^ | **0.047** |
| *sirt2* | 0.26±0.01^a^ | 0.21±0.01^b^ | 0.22±0.01^b^ | **0.018** |  | 0.15±0.00 | 0.14±0.01 | 0.15±0.01 | 0.842 |  | 0.41±0.07^a^ | 0.24±0.01^b^ | 0.36±0.04^a^ | **0.026** |
| *gpx4* | 0.28±0.07^a^ | 0.09±0.04^b^ | 0.13±0.04^ab^ | **0.041** |  | 0.16±0.09 | 0.13±0.04 | 0.09±0.03 | 0.854 |  | 0.87±0.21 | 0.80±0.22 | 0.47±0.09 | 0.437 |
| *gr* | 0.18±0.01^a^ | 0.13±0.01^b^ | 0.14±0.01^b^ | **0.008** |  | 0.09±0.01^a^ | 0.08±0.00^ab^ | 0.06±0.00^b^ | **0.004** |  | 0.31±0.07 | 0.16±0.01 | 0.24±0.03 | 0.078 |
| *mn-sod / sod2* | 1.56±0.07^a^ | 1.17±0.06^b^ | 1.56±0.08^a^ | **<0.001** |  | 1.28±0.11 | 1.18±0.15 | 0.95±0.06 | 0.100 |  | 1.97±0.14^ab^ | 1.60±0.08^b^ | 2.16±0.15^a^ | **0.009** |
| *grp170* | 0.20±0.01 | 0.24±0.02 | 0.18±0.02 | 0.052 |  | 0.15±0.02^a^ | 0.13±0.01^ab^ | 0.11±0.01^b^ | **0.015** |  | 0.76±0.11 | 0.56±0.04 | 0.72±0.08 | 0.414 |
| *grp94* | 0.65±0.05 | 0.70±0.04 | 0.71±0.05 | 0.662 |  | 0.18±0.03^a^ | 0.17±0.01^a^ | 0.11±0.01^b^ | **0.005** |  | 0.95±0.25 | 0.50±0.04 | 0.63±0.07 | 0.383 |
| *grp75/mortalin* | 0.83±0.04^a^ | 0.69±0.02^b^ | 0.87±0.03^a^ | **<0.001** |  | 0.54±0.05^a^ | 0.50±0.03^ab^ | 0.41±0.02^b^ | **0.025** |  | 1.92±0.28^a^ | 1.10±0.06^b^ | 1.84±0.19^a^ | **0.002** |
| *capn1* | 0.92±0.06 | 0.90±0.05 | 0.94±0.07 | 0.902 |  | 0.31±0.02 | 0.26±0.03 | 0.26±0.02 | 0.199 |  | 0.59±0.04 | 0.51±0.03 | 0.55±0.05 | 0.366 |
| *capn2* | 0.78±0.07 | 0.66±0.07 | 0.70±0.06 | 0.493 |  | 0.24±0.05 | 0.19±0.02 | 0.18±0.01 | 0.854 |  | 0.52±0.06 | 0.47±0.04 | 0.41±0.03 | 0.334 |
| *capn3* | 1.01±0.09^b^ | 1.43±0.10^a^ | 1.30±0.08^ab^ | **0.017** |  | 1.03±0.15 | 0.97±0.20 | 0.82±0.09 | 0.453 |  | 2.97±0.44 | 2.60±0.25 | 2.99±0.38 | 0.606 |
| *cast* | 2.21±0.15 | 2.71±0.25 | 2.16±0.21 | 0.175 |  | 1.21±0.12 | 1.18±0.07 | 1.01±0.10 | 0.301 |  | 9.75±2.18^a^ | 4.90±0.31^b^ | 9.46±1.16^a^ | **0.003** |
| *ctsb* | 0.99±0.06 | 0.96±0.08 | 0.82±0.04 | 0.119 |  | 0.46±0.05 | 0.41±0.03 | 0.43±0.04 | 0.749 |  | 2.24±0.46 | 1.30±0.09 | 1.63±0.17 | 0.144 |
| *ctsd* | 0.16±0.02 | 0.12±0.01 | 0.12±0.01 | 0.070 |  | 0.07±0.01 | 0.06±0.01 | 0.06±0.00 | 0.404 |  | 0.44±0.06^a^ | 0.28±0.02^b^ | 0.39±0.02^a^ | **0.026** |
| *ctsl* | 2.70±0.18 | 2.80±0.19 | 2.28±0.12 | 0.062 |  | 1.52±0.13 | 1.40±0.10 | 1.22±0.14 | 0.270 |  | 7.02±2.12 | 3.67±0.59 | 5.89±0.73 | 0.158 |
| *ctss* | 0.27±0.03 | 0.21±0.02 | 0.22±0.01 | 0.194 |  | 0.08±0.02 | 0.08±0.01 | 0.07±0.01 | 0.828 |  | 0.28±0.03 | 0.24±0.08 | 0.28±0.04 | 0.850 |
| *cat* | 1.65±0.09 | 1.69±0.08 | 1.61±0.07 | 0.748 |  | 1.83±0.13 | 1.46±0.09 | 1.52±0.11 | 0.066 |  | 3.04±0.27 | 2.27±0.11 | 2.96±0.28 | 0.062 |
