# Supplementary material for: Physiologically mediated responses in gilthead sea bream (Sparus aurata) fed sustainable diets: seasonal growth under warming conditions
Source: Front Physiol. 2026 Jun 30;17:1860904. doi: 10.3389/fphys.2026.1860904 (PMC13392755; doi:10.3389/fphys.2026.1860904)
Supplement: Supplementary file 7 [file Table7.docx]

Supplementary Table 7. Relative gene expression of anterior intestine mRNA transcripts of fish fed three experimental diets (CTRL, PAP, ALT) along three periods of time (t_1_, t_2_, t_3_). Values are the mean ± SEM of 10-16 fish per experimental condition. All data from t_1_, t_2_ and t_3_ are in reference to the expression level of *ccr9* in fish from CTRL group of each time with an arbitrary value of 1. Different letters indicate statistically significant differences (Holm-Sidak post hoc test, *p* < 0.05). Differentially expressed genes are in bold.

|  | t_1_ | | |  |  | t_2_ | | |  |  | t_3_ | | |  |
| --- | --- | --- | --- | --- | --- | --- | --- | --- | --- | --- | --- | --- | --- | --- |
|  | CTRL | PAP | ALT | *p* |  | CTRL | PAP | ALT | *p* |  | CTRL | PAP | ALT | *P* |
| *pcna* | 2.12±0.20 | 1.92±0.17 | 2.20±0.12 | 0.483 |  | 2.47±0.20 | 2.34±0.31 | 2.73±0.56 | 0.713 |  | 2.34±0.22 | 2.86±0.29 | 2.36±0.33 | 0.349 |
| *hes1b* | 1.14±0.14^b^ | 1.71±0.26^ab^ | 2.05±0.16^a^ | **0.009** |  | 1.57±0.16 | 1.41±0.24 | 1.37±0.23 | 0.779 |  | 1.07±0.12^b^ | 1.06±0.12^b^ | 1.54±0.13^a^ | **0.014** |
| *klf4* | 0.62±0.12 | 0.67±0.14 | 0.79±0.09 | 0.656 |  | 0.92±0.13 | 0.98±0.19 | 0.83±0.18 | 0.818 |  | 1.03±0.15 | 1.01±0.11 | 1.17±0.14 | 0.656 |
| *cldn12* | 0.37±0.03 | 0.37±0.04 | 0.40±0.03 | 0.829 |  | 0.44±0.04 | 0.41±0.05 | 0.45±0.06 | 0.856 |  | 0.38±0.06^ab^ | 0.33±0.04^b^ | 0.45±0.02^a^ | **0.015** |
| *cldn15* | 18.20±2.74 | 17.89±2.05 | 20.24±1.78 | 0.723 |  | 17.70±1.29 | 18.06±2.43 | 22.91±3.00 | 0.236 |  | 19.29±3.09^ab^ | 16.78±2.16^b^ | 27.81±2.64^a^ | **0.009** |
| *cdh1* | 7.31±0.68 | 6.44±0.62 | 7.21±0.25 | 0.705 |  | 8.05±0.47 | 7.50±0.77 | 8.40±1.24 | 0.749 |  | 6.13±0.43^b^ | 6.35±0.39^b^ | 9.58±0.43^a^ | **<0.001** |
| *cdh17* | 29.23±2.82 | 30.01±2.90 | 32.79±1.91 | 0.596 |  | 29.54±1.96 | 31.42±3.45 | 30.27±3.12 | 0.892 |  | 26.26±2.40^b^ | 33.37±3.02^ab^ | 40.70±3.54^a^ | **0.011** |
| *tjp1* | 0.22±0.02 | 0.23±0.02 | 0.22±0.02 | 0.968 |  | 0.27±0.02 | 0.28±0.04 | 0.32±0.04 | 0.639 |  | 0.35±0.04^b^ | 0.36±0.04^b^ | 0.53±0.04^a^ | **0.006** |
| *dsp* | 3.60±0.35 | 3.23±0.18 | 3.58±0.42 | 0.922 |  | 4.08±0.23 | 3.87±0.25 | 4.78±0.32 | 0.064 |  | 5.95±0.89 | 5.16±0.21 | 5.15±0.29 | 0.810 |
| *cx32.2* | 44.91±2.32 | 49.12±3.47 | 44.77±2.58 | 0.475 |  | 41.58±5.57 | 44.57±4.47 | 57.08±7.82 | 0.183 |  | 43.46±3.96 | 41.76±2.34 | 49.37±3.58 | 0.219 |
| *cxadr* | 2.73±0.19 | 2.57±0.16 | 2.76±0.18 | 0.698 |  | 3.11±0.27 | 2.80±0.19 | 3.27±0.34 | 0.462 |  | 3.12±0.26^b^ | 3.12±0.16^b^ | 4.00±0.20^a^ | **0.005** |
| *alpi* | 83.27±10.35 | 90.86±7.64 | 75.45±9.15 | 0.498 |  | 53.09±7.08 | 60.21±8.04 | 73.27±14.97 | 0.382 |  | 31.78±3.69^b^ | 35.99±2.47^b^ | 57.83±6.43^a^ | **0.007** |
| *fabp1* | 54.69±6.67 | 49.96±2.24 | 53.30±4.74 | 0.921 |  | 64.36±4.11 | 60.10±6.04 | 63.60±8.51 | 0.867 |  | 60.50±5.51^b^ | 60.56±4.94^b^ | 108.69±13.59^a^ | **0.002** |
| *fabp2* | 167.90±48.47^ab^ | 146.65±36.18^b^ | 421.95±115.49^a^ | **0.029** |  | 244.02±59.41 | 309.08±85.99 | 262.66±74.93 | 0.821 |  | 163.22±25.93 | 166.44±25.10 | 117.64±24.49 | 0.155 |
| *fabp6* | 0.14±0.10 | 0.05±0.02 | 0.02±0.01 | 0.467 |  | 0.05±0.03 | 0.04±0.04 | 0.06±0.03 | 0.499 |  | 0.06±0.02 | 0.11±0.05 | 0.15±0.08 | 0.914 |
| *muc2* | 36.59±3.54 | 31.31±3.90 | 33.15±2.89 | 0.271 |  | 58.11±5.52^a^ | 48.99±6.42^ab^ | 35.81±4.78^b^ | **0.043** |  | 66.67±5.06^ab^ | 55.99±2.43^b^ | 84.85±8.62^a^ | **0.010** |
| *muc13* | 28.16±1.57 | 25.71±1.38 | 27.16±1.86 | 0.567 |  | 37.54±3.04 | 35.18±2.87 | 35.63±5.28 | 0.885 |  | 40.01±4.01^b^ | 39.39±1.87^b^ | 66.97±5.53^a^ | **<0.001** |
| *tnfα* | 0.11±0.01^b^ | 0.12±0.01^ab^ | 0.14±0.01^a^ | **0.013** |  | 0.10±0.01 | 0.14±0.04 | 0.15±0.01 | 0.264 |  | 0.14±0.02^ab^ | 0.10±0.01^b^ | 0.17±0.02^a^ | **0.024** |
| *il1β* | 0.13±0.03 | 0.16±0.02 | 0.14±0.03 | 0.348 |  | 0.07±0.01 | 0.08±0.02 | 0.09±0.01 | 0.500 |  | 0.04±0.00 | 0.04±0.00 | 0.05±0.00 | 0.072 |
| *il6* | 0.01±0.00 | 0.01±0.00 | 0.01±0.00 | 0.404 |  | 0.01±0.00 | 0.01±0.00 | 0.01±0.00 | 0.371 |  | 0.01±0.00 | 0.01±0.00 | 0.01±0.00 | 0.125 |
| *il7* | 0.24±0.02 | 0.31±0.02 | 0.28±0.03 | 0.155 |  | 0.31±0.01 | 0.34±0.02 | 0.38±0.02 | 0.082 |  | 0.27±0.02 | 0.30±0.01 | 0.32±0.01 | 0.104 |
| *il8* | 0.12±0.02^b^ | 0.15±0.02^b^ | 0.33±0.03^a^ | **<0.001** |  | 0.19±0.02 | 0.24±0.05 | 0.32±0.02 | 0.062 |  | 0.30±0.04^b^ | 0.20±0.02^b^ | 0.70±0.12^a^ | **<0.001** |
| *il10* | 0.07±0.01 | 0.07±0.00 | 0.06±0.00 | 0.267 |  | 0.09±0.01 | 0.11±0.03 | 0.10±0.02 | 0.789 |  | 0.08±0.01^b^ | 0.10±0.01^ab^ | 0.13±0.01^a^ | **0.018** |
| *il12β* | 0.34±0.03 | 0.33±0.02 | 0.39±0.04 | 0.384 |  | 0.37±0.04 | 0.39±0.04 | 0.39±0.09 | 0.593 |  | 0.17±0.02^b^ | 0.19±0.01^b^ | 0.31±0.02^a^ | **<0.001** |
| *il15* | 0.49±0.02 | 0.46±0.02 | 0.41±0.02 | 0.051 |  | 0.48±0.03 | 0.56±0.03 | 0.65±0.12 | 0.119 |  | 0.81±0.12^b^ | 0.63±0.03^b^ | 1.01±0.08^a^ | **0.002** |
| *il34* | 0.62±0.04 | 0.61±0.04 | 0.54±0.03 | 0.295 |  | 0.74±0.06 | 0.72±0.05 | 0.82±0.05 | 0.424 |  | 0.70±0.03^b^ | 0.77±0.03^b^ | 1.00±0.04^a^ | **<0.001** |
| *cd4-1* | 0.26±0.02 | 0.23±0.02 | 0.25±0.03 | 0.649 |  | 0.23±0.03 | 0.25±0.04 | 0.34±0.06 | 0.205 |  | 0.23±0.02^b^ | 0.26±0.02^b^ | 0.34±0.02^a^ | **0.005** |
| *cd8b* | 0.06±0.01 | 0.04±0.00 | 0.06±0.01 | 0.052 |  | 0.06±0.01 | 0.06±0.01 | 0.07±0.01 | 0.846 |  | 0.07±0.01^ab^ | 0.06±0.01^b^ | 0.08±0.01^a^ | **0.043** |
| *ccr3* | 0.51±0.04 | 0.45±0.09 | 0.41±0.04 | 0.140 |  | 0.57±0.05 | 0.70±0.07 | 0.55±0.08 | 0.228 |  | 0.76±0.10^ab^ | 0.63±0.04^b^ | 0.93±0.10^a^ | **0.029** |
| *ccr9* | 1.06±0.13 | 1.02±0.09 | 1.05±0.10 | 0.921 |  | 1.03±0.08 | 0.91±0.07 | 0.99±0.05 | 0.456 |  | 0.88±0.11^b^ | 1.00±0.07^ab^ | 1.25±0.10^a^ | **0.029** |
| *ccr11* | 2.70±0.22^a^ | 2.08±0.17^b^ | 2.64±0.17^a^ | **0.048** |  | 3.28±0.27 | 3.60±0.43 | 2.91±0.40 | 0.456 |  | 2.73±0.27^ab^ | 2.82±0.12^b^ | 3.86±0.32^a^ | **0.007** |
| *ck8/ccl20* | 1.64±0.21 | 2.35±0.33 | 2.76±1.65 | 0.694 |  | 6.06±1.13^a^ | 4.97±0.78^a^ | 1.52±0.22^b^ | **<0.001** |  | 3.94±0.52^b^ | 6.56±0.65^a^ | 3.34±0.42^b^ | **<0.001** |
| *csf1r1* | 0.15±0.01 | 0.15±0.02 | 0.15±0.02 | 0.956 |  | 0.19±0.04 | 0.22±0.03 | 0.21±0.02 | 0.784 |  | 0.37±0.03^b^ | 0.38±0.02^b^ | 0.52±0.05^a^ | **0.009** |
| *igm* | 4.85±1.84 | 2.54±0.53 | 2.43±0.59 | 0.412 |  | 3.07±0.73^b^ | 3.08±0.63^b^ | 8.38±1.66^a^ | **0.002** |  | 4.68±1.05^b^ | 6.24±1.76^b^ | 12.66±2.37^a^ | **0.006** |
| *igt-m* | 0.62±0.22 | 0.62±0.17 | 0.67±0.17 | 0.432 |  | 0.53±0.11 | 0.53±0.09 | 0.58±0.11 | 0.772 |  | 0.43±0.05^b^ | 0.59±0.09^ab^ | 1.06±0.24^a^ | **0.015** |
| *lgals1* | 11.29±0.83 | 13.04±1.01 | 9.58±1.31 | 0.090 |  | 7.63±1.06 | 6.26±0.75 | 7.74±1.57 | 0.607 |  | 2.93±0.53^ab^ | 2.22±0.11^b^ | 3.67±0.32^a^ | **<0.001** |
| *lgals8* | 2.09±0.12 | 1.89±0.06 | 2.18±0.19 | 0.348 |  | 2.73±0.22 | 2.26±0.18 | 2.57±0.19 | 0.208 |  | 2.57±0.16^b^ | 2.37±0.11^b^ | 3.43±0.21^a^ | **<0.001** |
| *tlr2* | 0.41±0.03 | 0.43±0.03 | 0.38±0.03 | 0.521 |  | 0.34±0.03 | 0.41±0.04 | 0.41±0.03 | 0.352 |  | 0.49±0.06^b^ | 0.42±0.03^b^ | 0.63±0.05^a^ | **0.004** |
| *tlr5* | 0.03±0.00^ab^ | 0.04±0.00^a^ | 0.02±0.00^b^ | **0.038** |  | 0.02±0.00 | 0.02±0.00 | 0.02±0.00 | 0.167 |  | 0.02±0.00^ab^ | 0.01±0.00^b^ | 0.02±0.00^a^ | **0.016** |
| *tlr9* | 0.03±0.00 | 0.03±0.00 | 0.03±0.00 | 0.428 |  | 0.04±0.00 | 0.03±0.01 | 0.04±0.01 | 0.755 |  | 0.04±0.00^b^ | 0.05±0.00^b^ | 0.07±0.01^a^ | **0.008** |
| *cd209d* | 0.08±0.01^b^ | 0.11±0.01^a^ | 0.09±0.01^ab^ | **0.022** |  | 0.12±0.01 | 0.12±0.01 | 0.10±0.02 | 0.267 |  | 0.11±0.01^b^ | 0.12±0.01^b^ | 0.16±0.01^a^ | **0.007** |
| *cd302* | 7.19±0.51 | 7.26±0.37 | 7.14±0.24 | 0.975 |  | 7.45±0.41 | 7.16±0.36 | 7.70±0.73 | 0.747 |  | 5.86±0.45^b^ | 5.60±0.17^b^ | 7.05±0.32^a^ | **0.004** |
| *mrc1* | 0.81±0.04 | 0.81±0.10 | 0.64±0.04 | 0.150 |  | 0.77±0.09 | 0.91±0.07 | 0.62±0.07 | 0.068 |  | 0.65±0.06^b^ | 0.63±0.03^b^ | 0.91±0.08^a^ | **0.001** |
| *fcl* | 8.16±3.26 | 6.55±2.21 | 5.45±3.40 | 0.377 |  | 5.05±1.58 | 5.08±1.71 | 12.93±4.86 | 0.426 |  | 68.31±17.59 | 42.14±7.28 | 60.04±15.62 | 0.582 |
