# Supplementary material for: Physiologically mediated responses in gilthead sea bream (Sparus aurata) fed sustainable diets: seasonal growth under warming conditions
Source: Front Physiol. 2026 Jun 30;17:1860904. doi: 10.3389/fphys.2026.1860904 (PMC13392755; doi:10.3389/fphys.2026.1860904)
Supplement: Supplementary file 8 [file SupplementaryFile1.docx]

**Supplementary Figure 1.** Daily mean temperature time series and photoperiod from January 2020 to November 2025, illustrating the temporal dynamics of the thermal regime.
