# Supplementary material for: Physiologically mediated responses in gilthead sea bream (Sparus aurata) fed sustainable diets: seasonal growth under warming conditions
Source: Front Physiol. 2026 Jun 30;17:1860904. doi: 10.3389/fphys.2026.1860904 (PMC13392755; doi:10.3389/fphys.2026.1860904)

**Supplementary Figure 2.** (A) Liver histology of fish fed the CTRL diet, the PAP diet and the ALT diet at the three sampling points (t1, July 2022; t2, November 2022; t3, February 2023). An increase in hepatocyte volume due to intracellular fat deposition is evident from t1 onward across all dietary treatments. Giemsa staining (1st, 3rd, 5th column) and PAS staining (2nd, 4th, 6th column). Scale bars = 20 µm. Histological scoring of hepatic lipid (B) and glycogen (C) storage in fish fed with control (CTRL, red), processed animal protein (PAP, green) and alternative (ALT, black) diets along the feeding trial (from July 2022 to February 2023). Mean semiquantitative scoring (+ SEM) ranges from 0 (absence) to 3 (pervasive). Different letters indicate significant differences among dietary treatments within each timing (P < 0.05).


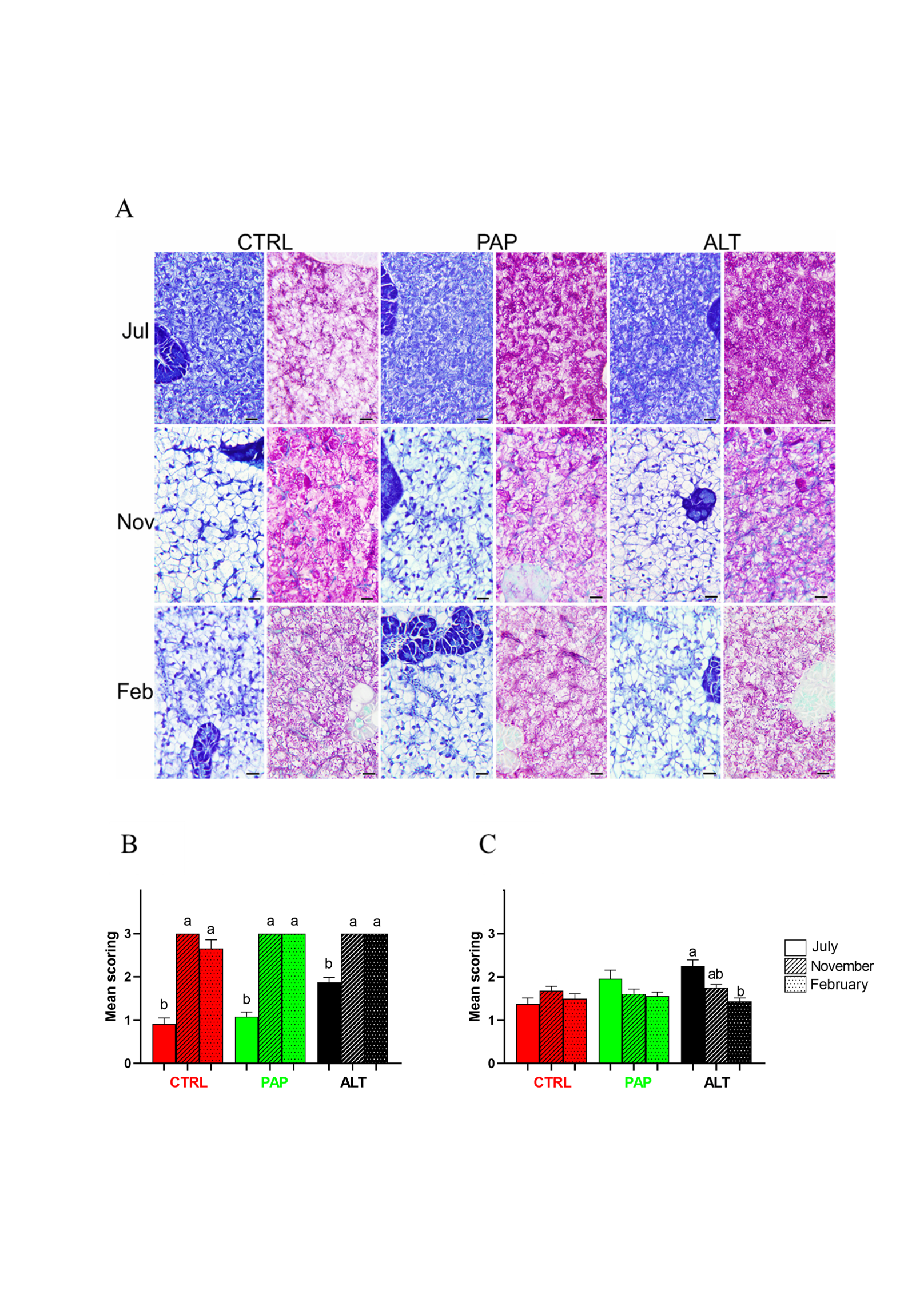

Supplement: Supplementary file 9 [file SupplementaryFile2.docx]
