# Supplementary material for: Physiologically mediated responses in gilthead sea bream (Sparus aurata) fed sustainable diets: seasonal growth under warming conditions
Source: Front Physiol. 2026 Jun 30;17:1860904. doi: 10.3389/fphys.2026.1860904 (PMC13392755; doi:10.3389/fphys.2026.1860904)

**Supplementary Figure 3.** (A) Anterior intestine histology of fish fed the CTRL diet, the PAP diet and the ALT diet at the three sampling points (t1, July 2022; t2, November 2022; t3, February 2023). No histopathological signs and no differences are observed among diets. Giemsa staining. Scale bars = 50 µm. Histological scoring at the anterior intestine of goblet cell abundance (B) and lipid vacuolization in enterocytes (C) in fish fed with control (CTRL, red), processed animal protein (PAP, green) and alternative (ALT, black) diets along the feeding trial (from July 2022 to February 2023). Mean semiquantitative scoring (+ SEM) ranges from 0 (absence) to 3 (very abundant). Different letters indicate significant differences among dietary treatments within each timing (P < 0.05).


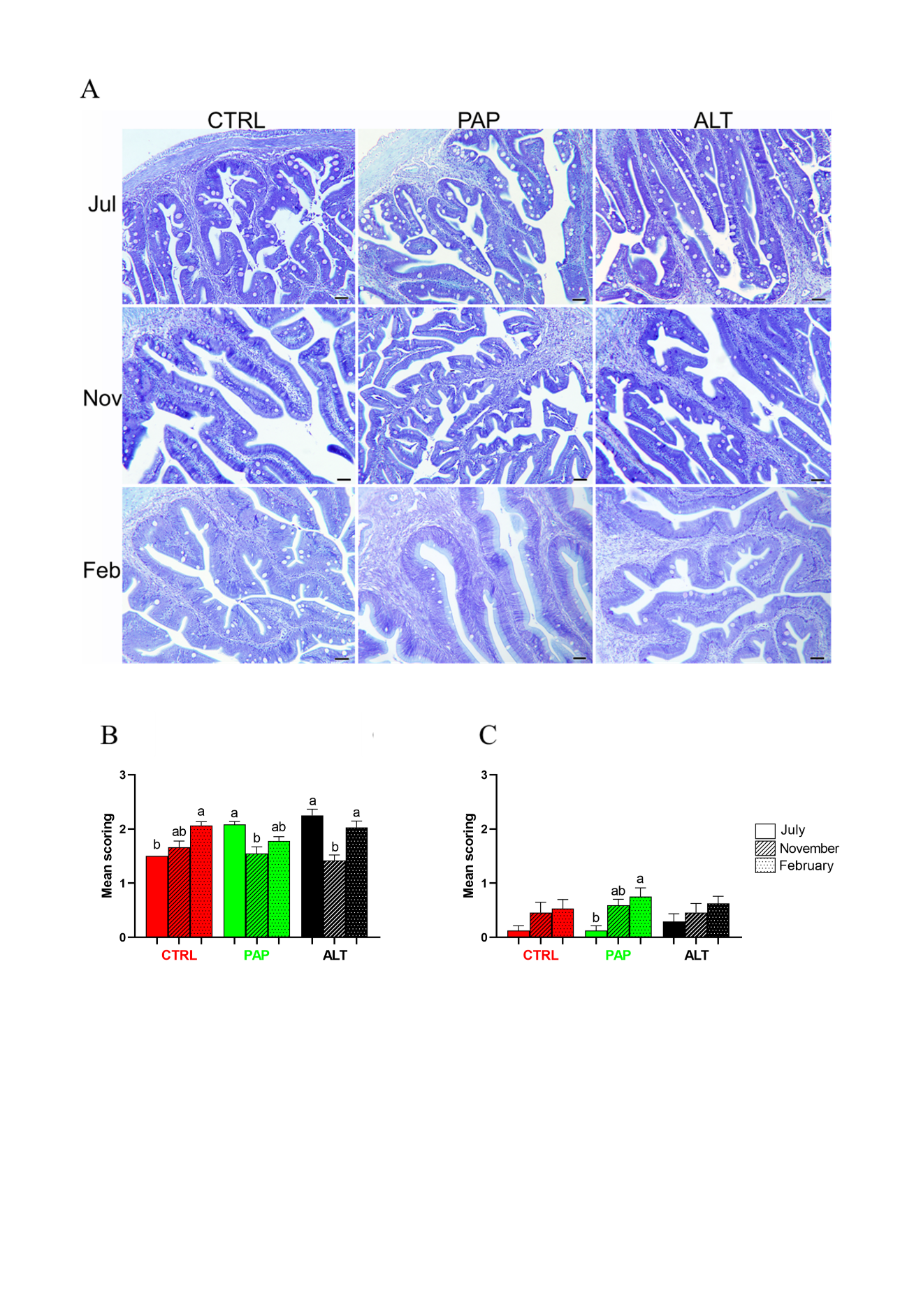

Supplement: Supplementary file 10 [file SupplementaryFile3.docx]
