# Supplementary material for: Physiologically mediated responses in gilthead sea bream (Sparus aurata) fed sustainable diets: seasonal growth under warming conditions
Source: Front Physiol. 2026 Jun 30;17:1860904. doi: 10.3389/fphys.2026.1860904 (PMC13392755; doi:10.3389/fphys.2026.1860904)

**Supplementary Figure 4.** (A) Posterior intestine histology of fish fed the CTRL diet, the PAP diet and the ALT diet at two sampling points (t2, November 2022; t3, February 2023). No histopathological signs and no differences are observed among diets. In t3, a decrease of goblet cell numbers and a slight hyperplasia of the intestinal submucosa was observed. Giemsa staining. Scale bars = 50 µm. Histological scoring at the posterior intestine of goblet cell abundance (B) and submucosal hyperplasia (C) in fish fed with control (CTRL, red), processed animal protein (PAP, green) and alternative (ALT, black) diets along the feeding trial (from November 2022 to February 2023). Mean semiquantitative scoring (+ SEM) ranges from 0 (absence) to 3 (very abundant/severe). Different letters indicate significant differences among dietary treatments within each timing (P < 0.05).


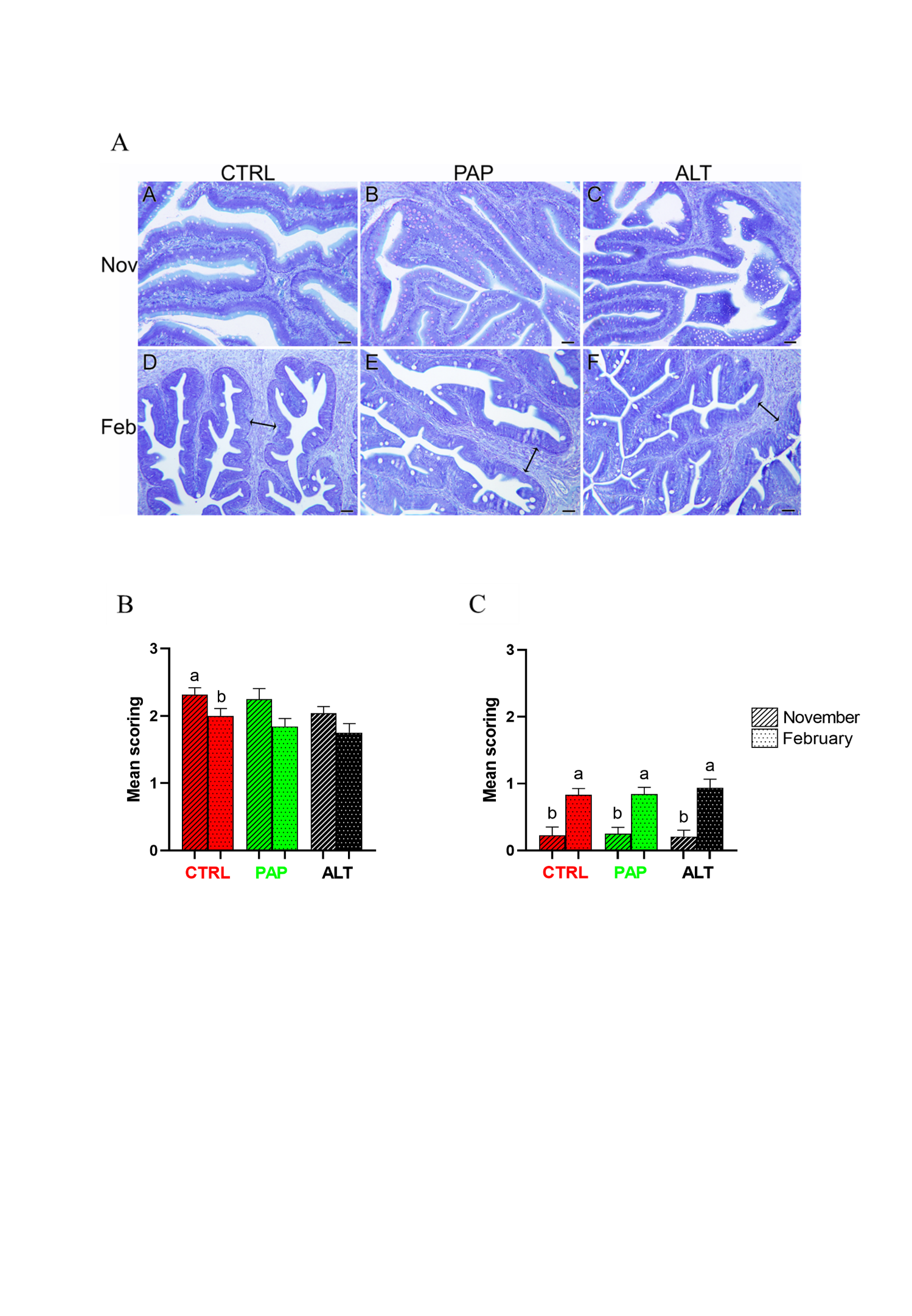

Supplement: Supplementary file 11 [file SupplementaryFile4.docx]
