# Supplementary material for: Physiologically mediated responses in gilthead sea bream (Sparus aurata) fed sustainable diets: seasonal growth under warming conditions
Source: Front Physiol. 2026 Jun 30;17:1860904. doi: 10.3389/fphys.2026.1860904 (PMC13392755; doi:10.3389/fphys.2026.1860904)
Supplement: Supplementary file 12 [file SupplementaryFile5.docx]

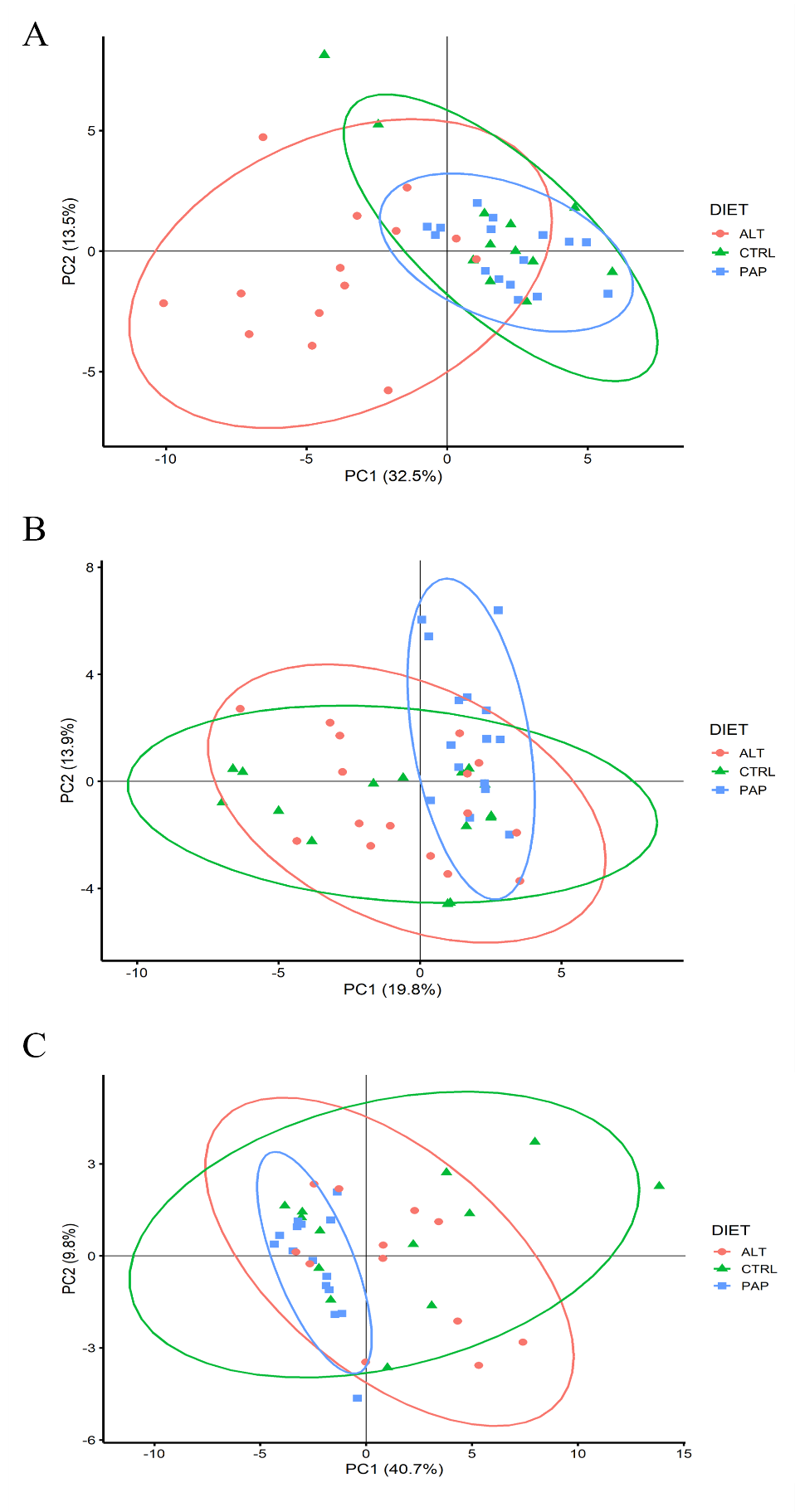
**Supplementary Figure 5.** Sample distribution across the first two components of Principal Component Analysis (PCA) plots among the three experimental groups (CTRL, ALT, and PAP) of the gene expression profiles at the T3, for AI (A), liver (B), and WSM (C). The percentage of variance explained by each component is reported on the axes, and confidence ellipses indicate the multivariate dispersion of samples within each group.
