# Supplementary material for: Physiologically mediated responses in gilthead sea bream (Sparus aurata) fed sustainable diets: seasonal growth under warming conditions
Source: Front Physiol. 2026 Jun 30;17:1860904. doi: 10.3389/fphys.2026.1860904 (PMC13392755; doi:10.3389/fphys.2026.1860904)
Supplement: Supplementary file 13 [file SupplementaryFile6.docx]

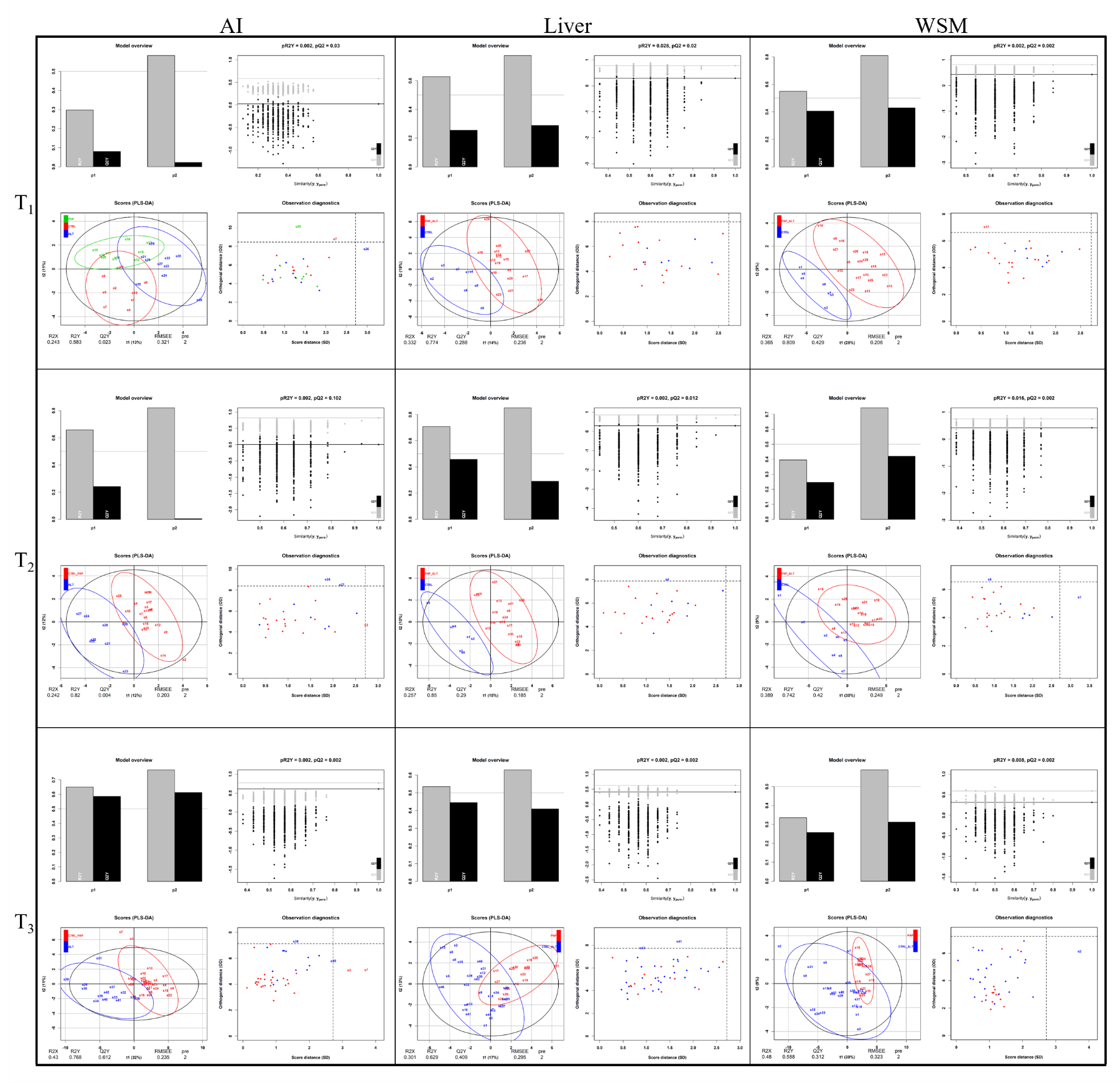
**Supplementary Figure 6.** Graphical representation of the validation for the PLS-DA models shown in Figures 5-7 corresponding to AI, liver and WSM at the three sampling points (T1-T3). The panel includes the contribution of each component to variance explained (R2Y) and predicted (Q2), the permutation plots (also considered a 7-fold cross-validation), score plot and the outliers identification.
