# Supplementary material for: Physiologically mediated responses in gilthead sea bream (Sparus aurata) fed sustainable diets: seasonal growth under warming conditions
Source: Front Physiol. 2026 Jun 30;17:1860904. doi: 10.3389/fphys.2026.1860904 (PMC13392755; doi:10.3389/fphys.2026.1860904)

**Supplementary Figure 7.** Physical activity before, during and after the stress test of gilthead sea bream fed (A) CTRL, (B) PAP and (C) ALT diet. Respiratory frequency before, during and after the stress test of gilthead sea bream fed (D) CTRL, (E) PAP and (F) ALT diet. AEFishBIT data (measures taken every 5min) of representative individuals (n = 6) is shown as a continuous dotted line in each panel. Gray shaded areas represent the confinement stress test.


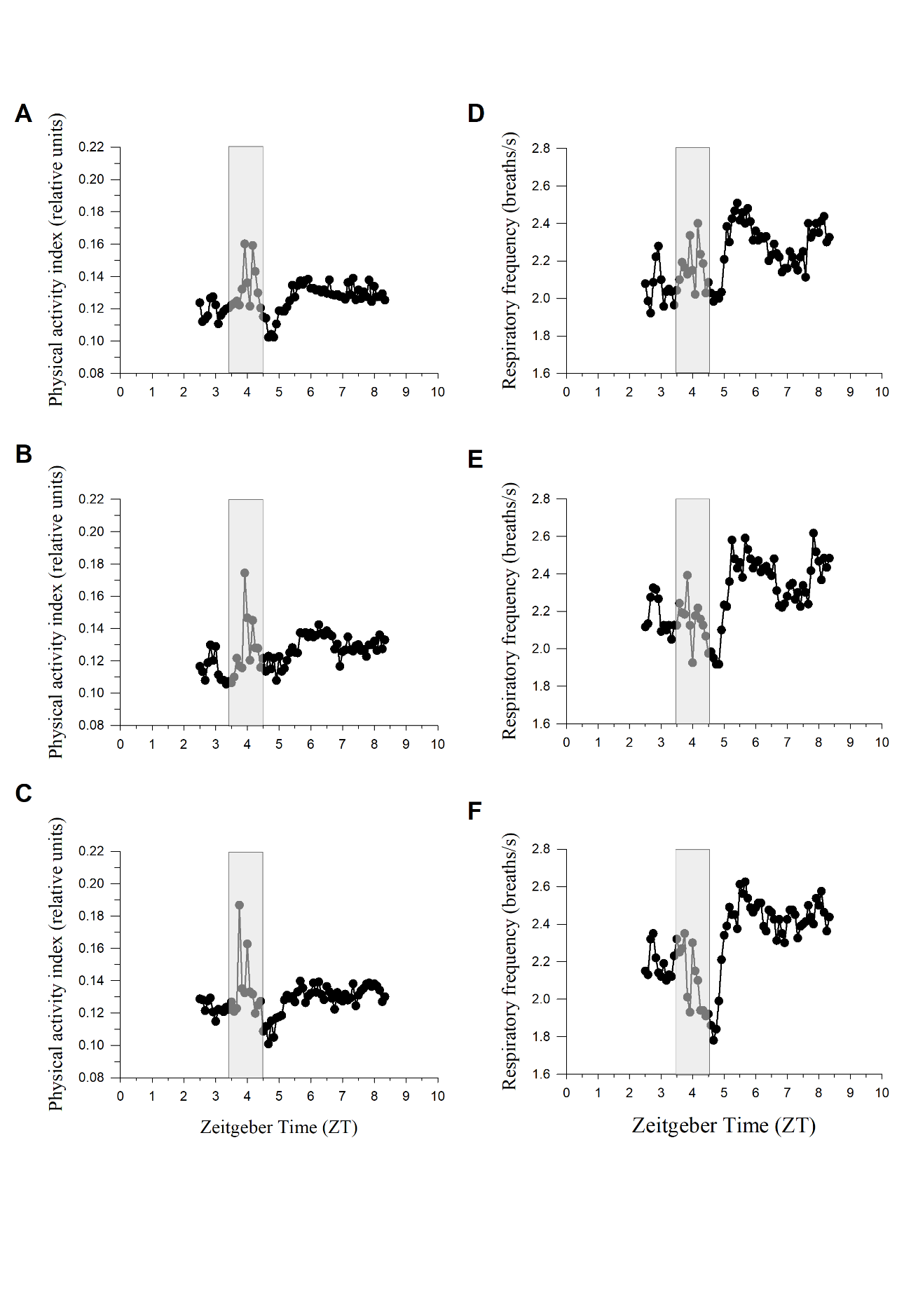

Supplement: Supplementary file 14 [file SupplementaryFile7.docx]
